# DNFE: Directed network flow entropy for detecting tipping points during biological processes

**DOI:** 10.1371/journal.pcbi.1013336

**Published:** 2025-07-29

**Authors:** Xueqing Peng, Rui Qiao, Peiluan Li, Luonan Chen

**Affiliations:** 1 School of Mathematics and Statistics, Henan University of Science and Technology, Luoyang, China; 2 Longmen Laboratory, Luoyang, Henan, China; 3 School of Mathematical Sciences and School of AI, Shanghai Jiao Tong University, Shanghai, China; 4 Guangdong Institute of Intelligence Science and Technology, Hengqin, China; 5 Key Laboratory of Systems Health Science of Zhejiang Province, Hangzhou Institute for Advanced Study, University of Chinese Academy of Sciences, Hangzhou, China; Arizona State University, UNITED STATES OF AMERICA

## Abstract

Typically, in dynamic biological processes, there is a critical state or tipping point that marks the transition from one stable state to another, surpassing which a considerable qualitative shift takes place. Identifying this tipping point and its driving network is essential to avert or delay disastrous outcomes. However, most traditional approaches built upon undirected networks still suffer from a lack of robustness and effectiveness when implemented based on high-dimensional small-sample data, especially for single-cell data. To address this challenge, we develop a directed network flow entropy (DNFE) method, which can transform measured omics data into a directed network. This method is applicable to both single-cell RNA-sequencing (scRNA-seq) and bulk data. Applying this algorithm to six real datasets, including three single-cell datasets, two bulk tumor datasets, and a blood dataset, the method is proved to be effective not only in identifying critical states, as well as their dynamic network biomarkers, but also in helping explore regulatory relationships between genes. Numerical simulation results demonstrate that the DNFE algorithm is robust across various noise levels and outperforms existing methods in detecting tipping points. Furthermore, the numerical simulations for 100-node and 1000-node gene regulatory networks illustrate the method’s application for large-scale data. The DNFE method predicts active transcription factors, and further identified “dark genes”, which are usually overlooked with traditional methods.

## Author summary

Numerous sophisticated systems undergo critical transitions, resulting in a sudden shift from one state to a markedly different state. Typically, in dynamic biological processes, there is a critical state or tipping point that marks the transition from one stable state to another, surpassing which a considerable qualitative shift takes place. Identifying this tipping point and its driving network is essential to avert or delay disastrous outcomes. However, most traditional approaches built upon undirected networks still suffer from a lack of robustness and effectiveness when implemented based on high-dimensional small-sample data, especially for single-cell data. To address this challenge, we develop a directed network flow entropy (DNFE) method, which can transform measured omics data into a directed network. This method is applicable to both single-cell RNA-sequencing (scRNA-seq) and bulk data. Applying this algorithm to six real datasets, including three single-cell datasets, two bulk tumor datasets, and a blood dataset, the method is proved to be effective not only in identifying critical states, as well as their dynamic network biomarkers, but also in helping explore regulatory relationships between genes. Our results suggest that the DNFE method predicts active transcription factors, and further identified “dark genes”, which are usually overlooked with traditional methods.

## Introduction

Numerous sophisticated systems undergo critical transitions, resulting in a sudden shift from one state to a markedly different state [1]. Single-cell RNA sequencing has revolutionized the investigation of cellular heterogeneity and functional diversity, enabling gene expression measurements within individual cells and allowing for inferences of cell population arrangements based on the trajectory topology and gene regulatory analysis [2–4]. A critical state exists in many biological processes, such as cell differentiation or embryonic development, where a marked transition or change in cell populations takes place [5–8]. Detecting a tipping point immediately prior to such critical transitions during biological processes is essential for shedding light on the intrinsic mechanisms involved in disease advancement [9,10]. However, owing to the similarity between the normal state and critical state, with respect to phenotype, along with average gene expression, traditional biomarkers often fail to detect these critical states.

To indicate the tipping point preceding the transition of biological systems, a theoretical framework referred to as the dynamic network biomarker (DNB) was introduced [11]. Based on the DNB theory, numerous methods have been proposed and utilized to identify the tipping points of complex diseases, such as influenza [12], breast cancer [13], and bladder urothelial carcinoma [14], as well as cell differentiation [15,16]. However, the use of this approach in single-cell RNA-sequencing (scRNA-seq) data analysis is limited because of obvious disturbances from amplification noise in transcripts and the occurrence of dropout events. Moreover, most of these computational approaches merely identify correlations among molecules/genes based on undirected networks, yet directed networks can reflect the interactions among genes and mine the underlying dynamic information in cell populations. In addition, directed networks can disclose the fundamental mechanisms underlying molecular effects, which provides quantitative data to support further precise therapies. Directed networks can explicitly represent directional relationships between genes, which is crucial for describing causal relationships. Directed networks can also gain a better understanding of the regulatory relationships between genes, and identifying important regulatory factors and pathways. Numerous GRN inference methods based on directed networks have also been developed, as highlighted in recent reviews [17]. For instance, ANANSE [18] prioritizes static regulatory edges via a network-based method that uses properties of enhancers and their GRNs to predict key TFs in cell fate determination, and DeepMAPS [19] leverages building a heterogeneous graph containing both cells and genes. While these methods effectively identify gene-gene interactions, none yield similar instability scores. Thus, proposing a resilient and effective method is urgent for detecting critical states based on directed networks, especially for single-cell data from a complex biological process.

To address these issues, we developed a novel computational algorithm based on directed networks, called the directed network flow entropy (DNFE) method, to predict critical transitions in bulk and single-cell data. Unlike conventional GRN inference approaches, our method first constructed a time-specific directed network at a given time point. We utilize the DNFE scores as instability scores to describe the molecular collective fluctuation qualify the criticality of the biological process (The detailed differences are presented in S1 Table). Differing from conventional methods, we prioritize the first-order neighbors that have a direct connection to the core gene, in conjunction with the second-order neighbors that directly interact with any first-order neighbors. Specifically, we first construct a time-specific directed network at a given time point with a direction determination index. We then calculate the local DNFE for each local directed network according to the information of the directed network. Unlike traditional information entropy, DNFE fully utilizes local (core gene) network connectivity information rather than fluctuations in gene expression, thereby reliably quantifying network fluctuation. Finally, we utilize the DNFE score to describe the molecular collective fluctuation resulting from specific samples against the reference samples/cells and qualify the criticality of the biological process, i.e., critical collective fluctuation. DNFE is an effective means of identifying the tipping points during the course of complex diseases, and it has the following advantages: (i) The DNFE method decreases noise by leveraging dynamic and high-dimensional information from omics data through the reliable quantification of critical network fluctuations, thereby increasing its robustness. Moreover, we focus on the second-order neighbors, which better depict the structure of the network, thereby improving the efficiency of the method. The numerical simulation shows the algorithm’s robustness, effectiveness, and applicability in handling vast amounts of data or large-scale datasets. (ii) Unlike most traditional methods, the DNFE method is proposed based on directed networks, which can help us gain a better understanding of the regulatory relationships between genes, identify important regulatory factors and pathways, and explore the structure and functionality of a gene regulatory network. (iii) The DNFE method can detect critical states before state transitions occur and recognize relevant DNB members with critical collective fluctuations and non-differential “dark genes”, potentially serving as prognostic biomarkers of complex diseases and biological processes. (iv) The DNFE method can not only be applied to bulk and scRNA-seq data but can also be used to identify transcription factors (TFs) that serve as fundamental players influencing cell identity and guiding shifts in cell fate as well. One such identified TF is *ZNF888*, which is considered to hold great potential in early cell differentiation.

To illustrate the resilience and efficiency of DNFE, we conducted numerical simulations using gene expression data from an artificial gene regulatory network, varying the noise strength. With the increase in noise strength, DNFE outperformed existing methods [20–23] in detecting early-warning signals for impending critical states. Furthermore, the numerical simulation, conducted on a 1000-node network, unequivocally showed the algorithm’s robustness and applicability in handling vast amounts of data or large-scale datasets. The DNFE method was applied to two bulk sequencing tumor datasets, including kidney renal papillary cell carcinoma (KIRP) and bladder cancer (BLCA) from The Cancer Genome Atlas (TCGA) database, effectively identifying critical states. Furthermore, cell fate commitment was accurately observed in three single-cell datasets, including the differentiation of human embryonic stem cells (hESCs) into definitive endoderm cells (DECs), mouse ESCs (mESCs) into mesoderm progenitor (MP) cells, and mouse embryonic fibroblasts (MEFs) into neurons. DNFE identified two critical states (at 12 and 36 h of differentiation) in the differentiation process data for DECs. The tipping points at 12 and 36 h may represent the critical points of ES-to-ME and ME-to-DE transitions. Finally, the DNFE method was applied to a blood dataset of patients with a non-complicated SLE pregnancy (SLE-NC). Immunological changes in neutrophils and T cells at five time points were detected, which aligns with the transcriptional changes surrounding embryo implantation monitored during assisted reproductive technology, thereby demonstrating the efficacy of DNFE.

## Results

### Validation based on numerical simulation

**Fig 1:**
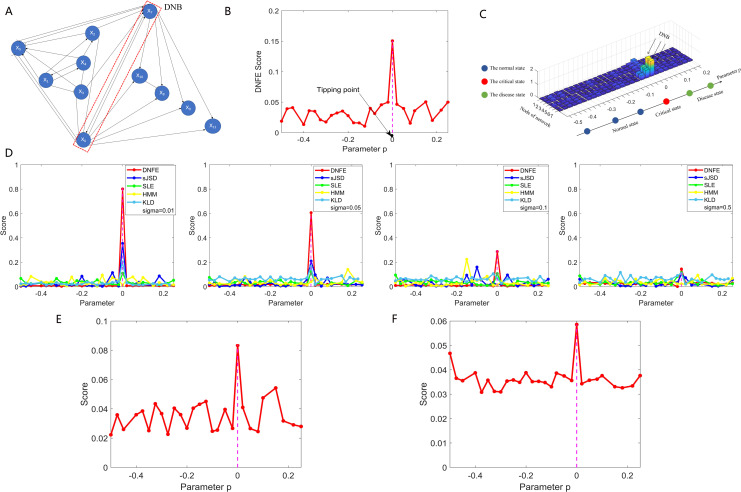
Validation of the directed network flow entropy (DNFE) method based on simulation datasets. (A) The 11-node network model depicts positive regulation with arrows and negative regulation with solid lines. (B) The curve of the DNFE score derived from the gene regulatory network. (C) The landscape of the DNFE score for 11 nodes. (D) Assessment of the stability of DNFE compared to alternative methods with varying noise intensities. (E) DNFE score curve based on the 100-node gene regulatory network. (F) DNFE curve score based on the 1000-node gene regulatory network.

To evaluate the efficacy and resiliency of the developed DNFE method, we used an eleven-node artificial network (Fig 1A) to illustrate the detection of early-warning signals when the system nears a critical state. Models based on Michaelis–Menten kinetics or Hill equations are widely used to describe the dynamics within gene regulatory networks [23–25], including processes, like transcription and translation [26,27], cyclic reactions [28], nonlinear biological processes [29,30], and other gene-regulatory activities [27,29]. A parameter varying between -0.5 and 0.23, with a bifurcation parameter value identified as the tipping point, was used to generate two simulated datasets.

Fig 1B depicts the dynamic variation in the DNFE score for the 11-node network. A marked increase in the DNFE score occurs at a specific parameter value, signaling an upcoming critical state. To effectively demonstrate the difference between the normal state and the pre-disease state, the local DNFE scores for the 11 individual networks are presented in Fig 1C, showcasing the overall landscape of the network flow entropy. Notably, as the system is distant from the critical state, the DNFE scores for all nodes remain stable and low. However, as the system nears the critical state, certain nodes exhibit abruptly altered expression variations and network connections (i.e., DNB), leading to a pronounced surge in the DNFE score, suggesting an impending critical state.

To ascertain the method’s robustness, as depicted in Fig 1D, we compared the DNFE algorithm with established DNB methods across varying levels of noise. With an escalating intensity of noise, the DNFE method outperforms the existing methods, such as single-sample-based Jensen-Shannon Divergence (sJSD) method [20], single-sample landscape entropy (SLE) method [21], hidden Markov model (HMM) method [22], and Kullback-Leibler divergence (KLD) method [23] in stably delivering early-warning signals of critical transition, thereby verifying its superior efficacy and robustness.

In addition, two simulation datasets were generated using a 100-node and a 1000-node structure depicted by stochastic differential equations to detect early-warning signals of critical transitions in genetic regulatory networks. The dynamic changes in DNFE score for the 100-node network and the 1000-node network are illustrated Fig 1E and 1F, respectively. An abrupt increase in the DNFE score is observed at a specific parameter value p=0, indicating the proximity to a critical state at a bifurcation point (p=0). As the system nears the tipping point, an abrupt increase in the DNFE score is observed around the bifurcation point p=0. Notably, the DNFE method demonstrates robustness when tested with the even larger 1000-node network. Additionally, the application of the algorithm to the simulation dataset generated by the 1000-node network resulted in quick calculations, underscoring the DNFE method’s applicability for large-scale datasets. Detailed simulation and calculation information can be found in S2 File and S1 Fig.

### Predicting critical states for hESC-to-DEC

To depict the efficiency of the developed method for single-cell datasets, we utilized DNFE on three scRNA-seq datasets of cell differentiation: hESC-to-DEC data, mESC-to-MP data, and MEF-to-neuron data. The application of the DNFE method to hESC-to-DEC data is illustrated within the primary text, and the analytical results using mESC-to-MP data and MEF-to-neuron data are provided in S3 File, S2 Fig for mESC-to-MP and S3 Fig for MEF-to-neuron data.

**Fig 2:**
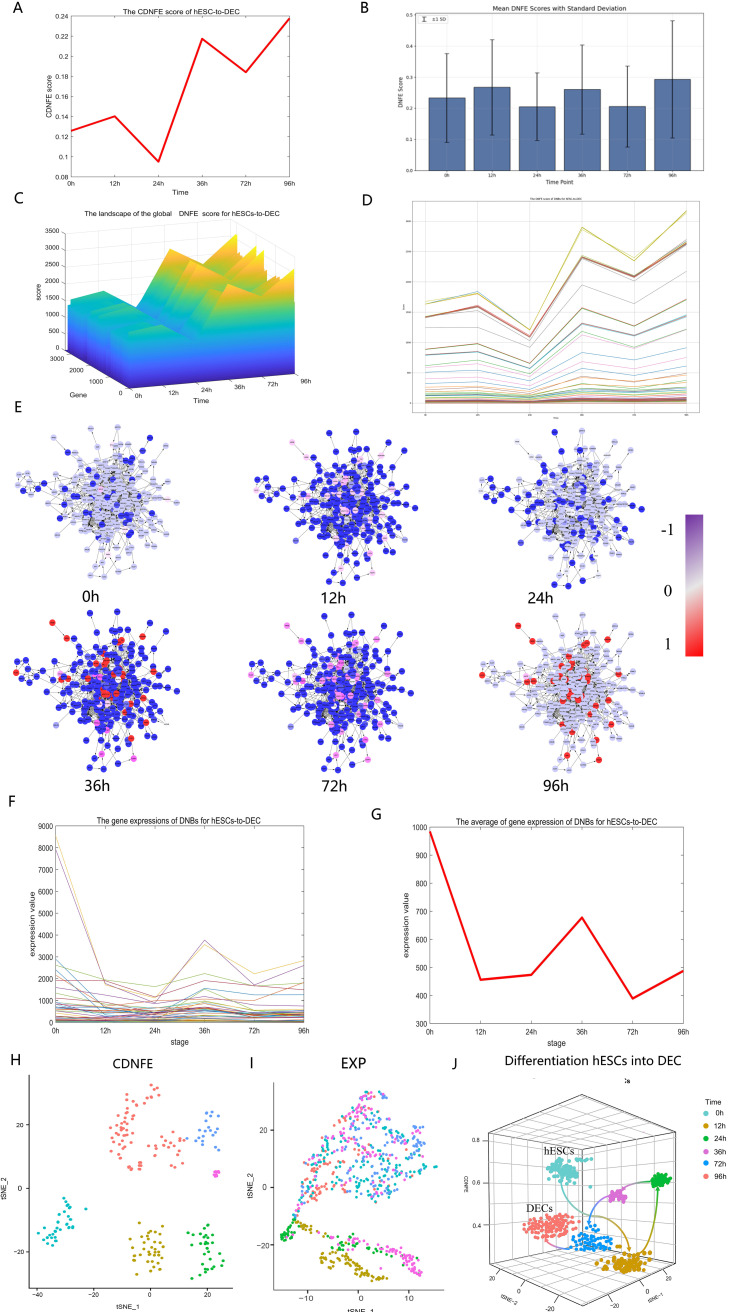
Critical states of the human embryonic stem cell (hESC) differentiation process revealed by directed network flow entropy (DNFE). (A)The DNFE scores at 12 and 36 h of hESC differentiation are elevated compared to those at the other time points, suggesting that these intervals represent critical states in the process of hESC differentiation. (B) The errorbars at each time point. (C) The landscape of the global DNFE score for hESC-to-definitive endoderm cell (DEC) transitions. The dynamic network biomarker (DNB) scores reach their peak at 12 h and 36 h, indicating the two tipping points. (D)The DNFE scores of DNBs for hESC-to-DEC transitions. (E) The gene-directed regulatory network’s dynamic evolution is established through signaling genes. The nodes and directed edges represent genes and the interactions between genes, respectively. (F) The gene expression of DNBs for hESC-to-DEC transitions. (G) The mean gene expression of DNBs for hESC-to-DEC transitions. Clearly, gene expression levels of DNBs are insufficient to differentiate the critical state from other states, while the DNFE method can successfully distinguish between them. (H)-(J) The nodes, represented in various colors, correspond to cells at different time points. The DNFE method is superior to the EXP method at distinguishing temporal cell states. Additionally, DNFE scores can provide accurate predictions of differentiation trajectories.

As depicted in Fig 2A, two critical states are identified by a sudden increase in the DNFE score: the first tipping point occurs at 12 h (P = 0.05) and the second at 36 h (P = 0.03). We have associated errorbars to Fig 2B to describe the changes in DNFE scores over time. As shown in this figure, the bar chart represents the average DNFE score at each time point, while the standard deviation (SD) reflects the dispersion of the data points. Calculations reveal that the standard deviation at each time point is predominantly within the range of 0.1 to 0.2. These transitions indicate two distinct differentiation processes following critical transitions, which are ES-to-ME and ME-to-DE. The top 5% of genes exhibiting the highest DNFE scores are designated as DNBs, and they are especially responsive to the tipping point that precedes disease deterioration. Fig 2C illustrates the landscape of the global DNFE score, where the score abruptly increases at 12 h and 36 h, suggesting identified tipping points or critical states. We visualized the score distribution for each gene, as illustrated in the Fig 2D, which shows the DNFE score abruptly increases at 12 h and 36 h. Fig 2E displays the dynamic development of the directed PPI network of DNFE signal biomarkers. There is an obvious and substantial shift in the DNFE score of the network following the critical point, suggesting that the network structure experiences a notable change. The nodes represent genes, and directed edges represent the interactions between genes. Moreover, as illustrated in Fig 2F and 2G, the gene expression levels of DNB genes alone do not effectively differentiate the critical state from other states. However, utilizing the DNFE scores of these DNB genes, the critical state can be identified successfully, as demonstrated in Fig 2A.

As our sample size met cluster analysis criteria (the sample size should be at least 5 times greater than the perplexity value, with a default value typically set at 30), we performed it to further demonstrate that DNFE can distinguish between different cellular states. The clustering analyses depicted in Fig 2H and 2I reveal that clustering analysis based on DNFE can effectively differentiate between cellular states at various time points, outperforming gene expression-based methods. The quantitative assessment of clustering performance is conventionally benchmarked using the Adjusted Rand Index (ARI). DNFE-based clustering demonstrated superior performance (ARI = 0.78) compared to expression-based clustering (ARI = 0.15). Network-based correlation works better than floating expression via gene expression. By employing the DNFE score, we can effectively mitigate the high noise and enhance the robustness of gene expression data. To further verify the capabilities of DNFE, pseudo-trajectory analysis was conducted on the hESC-to-DEC data. Performing temporal cell clustering via DNFE, three-dimensional representations of cell-lineage trajectories are presented in Fig 2H, where the z-axis signifies DNFE’s potency estimation, while the x-axes and y-axes correspond to the t-SNE components. Fig 2J showcases the differentiation trajectories of cells transitioning from hESCs to DECs, with differentiation towards DECs observed after 36 h, aligning with the experimental findings [31]. These findings underscore the capability of DNFE-based potency estimation to monitor dynamic changes in cell potency and pinpoint the precise time points of cell fate commitment or differentiation into various cell types.

### Identifying TFs in the endodermal differentiation of hESCs

**Fig 3:**
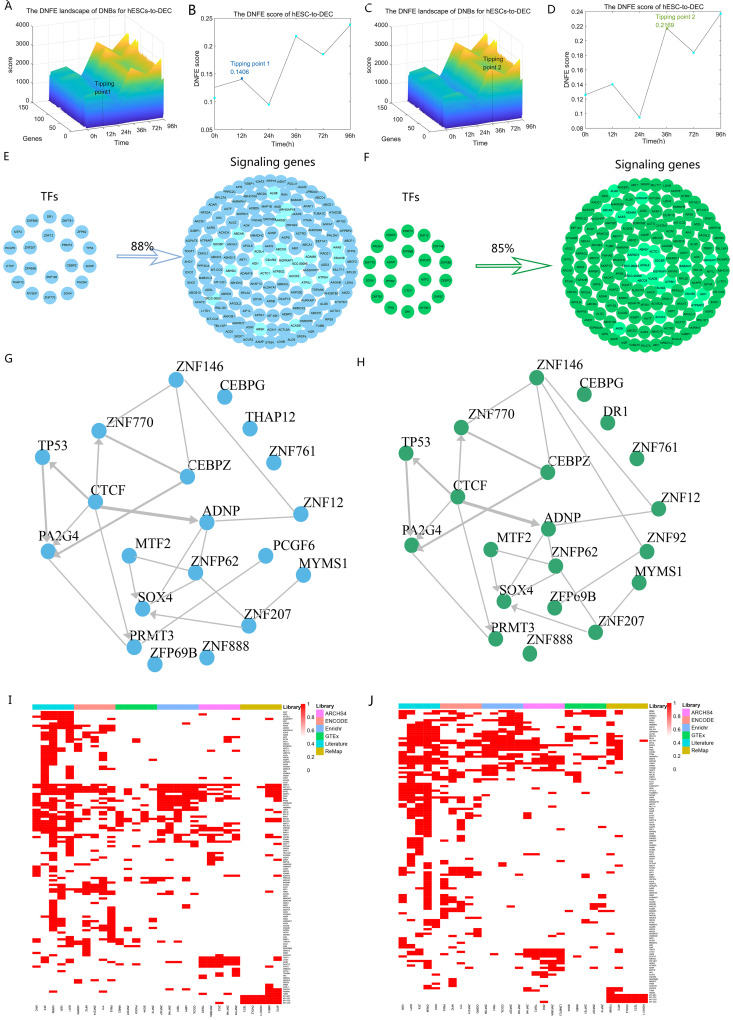
Critical states of human embryonic stem cell (hESC) differentiation process revealed using the directed network flow entropy (DNFE) method. (A) The expression dynamics of dynamic network biomarker (DNB) genes at the 12 h mark during hESC differentiation highlight the initial stages of cellular commitment. (B) The DNFE scores at 12h provide a quantitative measure of the systemic perturbations within the cellular network, indicative of the first critical transition. (C) The behavior of DNB genes at the 36h mark further delineates the gene expression patterns as differentiation progresses. (D) The DNFE scores at 36 h offer a subsequent quantitative assessment of network disruption, marking the second critical transition. (E) It was discovered that a mere twenty pivotal upstream transcription factors are capable of regulating a substantial 88% of the DNB genes identified at the first critical state (12 h), underscoring the efficiency of the regulatory network. (F) Similarly, at the second critical state (36 h), the same set of twenty transcription factors can modulate 85% of the DNB genes, demonstrating their sustained influence. (G) The regulatory interactions among the top 20 transcription factors at the first tipping point were analyzed, revealing the complex interplay that governs early differentiation events. (H) At the second tipping point, the regulatory relationships among the top 20 transcription factors were also examined, providing a deeper understanding of the later stages of cellular commitment. (I) The matrix representing the top 5 transcription factors from each library, aligned with query genes at the first tipping point, was constructed to show the presence of query genes within the target gene sets of library transcription factors. (J) A similar matrix was created for the second tipping point, illustrating the targeted gene regulation by the top 5 transcription factors, as identified using each library.

The top 5% of genes with the highest DNFE scores serve as DNBs. And the DNB genes do not demonstrate differential expression but are sensitive to DNFE scores being termed “dark genes”. As expected, the DNB genes at 12 h showed higher scores than those of the two surrounding time points (0 and 24 h) (Fig 3A and 3B), similarly to the DNB genes at 36 h (Fig 3C and 3D).

Next, we aimed to pinpoint the possible upstream transcriptional regulators of the DNB genes associated with the two critical states. We focused on TFs due to their crucial role in defining cell identity and driving cell fate transitions. We predicted the TFs on the CHEA3 website and identified 20 TFs for the first critical state (12 h) and 20 TFs for the second critical state (36 h) based on the chosen top 150 DNBs. These two groups of TFs could regulate 88% and 85% of the DNBs in each tipping point, respectively (Fig 3E and 3F). Among these DNB factors, *ZNF146* is associated with a poor cancer prognosis and functions as a pro-proliferative and growth-sustaining transcriptional regulator in cells, exerting an obvious effect on cellular dynamics [32]. *CEBPZ* is the key TF that regulates various aspects of cellular differentiation and functions in a variety of tissues [33]. The overexpression of *PRMT3* facilitates cell proliferation, migration, and invasion. Moreover, *PRMT3* stabilizes *C-MYC*, and the pro-proliferation role of *PRMT3* depends on *C-MYC* [34]. Within hESCs, *ZNF207* collaborates with key pluripotency TFs to regulate self-renewal and maintain pluripotency, while also directing cell commitment towards the ectoderm by directly regulating neuronal TFs, holding a crucial position in differentiation [35]. As demonstrated in Fig 3G, the local network shows the results of the mutual regulation between the predicted top 20 TFs in the regulatory network at the first tipping point, similarly to the second tipping point (Fig 3H). Particularly, among the forecasted top 20 TFs, *ZNF888* has been pinpointed as a methylation-influenced gene correlated with clear-cell renal cell carcinoma. It can enhance cellular proliferation through the suppression of *CDKN1A*, a gene that inhibits the cell cycle [36]. Nonetheless, given the scant research on *ZNF888*, its precise role remains elusive, necessitating additional investigations to fully elucidate its function in cellular processes. The heatmaps in Fig 3I and 3J display whether a gene is included in the target gene set of TFs from the library. The top five TFs are included in each library, and each column represents each TF. *SRSF3* and *HNRNPH1* appear most frequently in the target gene set of the TF library at the two tipping points. *SRSF3* is regarded as more important in the termination of transcription than in cleavage, potentially through its interaction with RNA sequences downstream of the cleavage site [37]. Both in vivo and in vitro studies have indicated that silencing *HNRNPH1* hinders cell proliferation and enhances apoptosis in Chronic Myeloid Leukemia cells [38].

In summary, using the DNFE method, we have successfully detected two critical states that occur in the differentiation of hESCs into the endoderm. Additionally, the analysis has led to the prediction of twenty TFs that can serve as pivotal regulators in determining cell fate during this differentiation process.

### Revealing functional roles of DNBs and “dark genes” for hESC-to-DEC

To delve deeper into the uncharted territory of regulatory elements in cancer-related pathways, as highlighted in [39], we conducted a comparative analysis between the DNB genes and the differentially expressed genes (DEGs). Our investigation revealed the presence of certain genes within the DNBs that did not exhibit differential expression at the molecular level but displayed elevated DNFE scores at the network level. These genes, termed “dark genes”, may have an apparent influence within the network context despite their lack of differential expression at the individual gene level.

**Fig 4:**
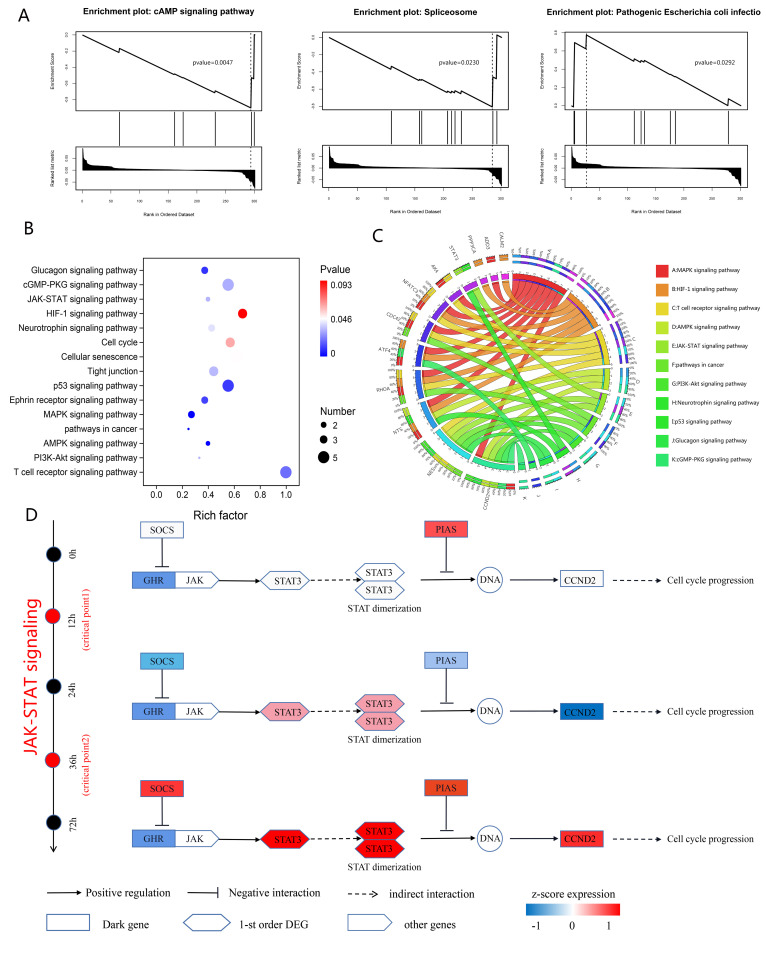
Regulatory mechanisms of embryo development revealed by the “dark genes” in the human embryonic stem cell (hESC)-to-definitive endoderm cell (DEC) process. (A) Gene set enrichment analysis of “dark genes”. (B) Enrichment analysis of Kyoto Encyclopedia of Genes and Genomes pathways for the “dark genes” in the hESC-to-DEC process. (C) The outer ring’s left segment delineates the dynamic network biomarkers (DNBs) recognized using the DNFE method, highlighting their presence in the differentiation process. The outer ring’s right segment corresponds to the array of biological processes in which these DNBs participate, showcasing their multifaceted roles. Within the inner ring, the color-coding and link thickness are utilized to represent the diversity of enriched pathways and the statistical significance of gene functions, respectively. (D) The “dark genes” and their first-order differentially expressed gene neighbors are further examined to uncover the intrinsic signaling mechanisms.

To identify important pathways to characterize the potential biological mechanisms of “dark genes” for hESC-to-DEC data, we next performed gene set enrichment analysis (GSEA). Detailed information from the hallmark pathway enrichment analysis is shown in S2 Table. As shown in Fig 4A, the crucial pathways included the cAMP signaling pathway, the spliceosome, and pathogenic *Escherichia coli* infection. In addition, we conducted Kyoto Encyclopedia of Genes and Genomes (KEGG) pathway enrichment analysis to uncover the potential biological functions of the “dark genes” and their first-order DEGs within the DNB genes, exhibiting molecular-level differential expression. The enrichment analysis revealed that these “dark genes” and their DEG counterparts were evidently enriched in key signaling pathways, such as JAK–STAT signaling, the PI3K–Akt signaling pathway, the cell cycle, and pathways in cancer, along with other cancer-related pathways (Fig 4B and 4C). Notably, the antagonism of JAK–STAT signaling has been shown to impede the progression of pre-neoplastic lesions to malignant tumors [40]. The PI3K–Akt signaling pathway has a key function in tumor cell differentiation, proliferation, and apoptosis [41]. Abnormal cell proliferation in cancer is associated with dysregulation of the cell cycle [42]. The involvement of multiple signaling “pathways in cancer” underscores its complexity and close relationship with the disease [43]. Aberrant regulation of the cell cycle is an important malignant characteristic of cancer, potentially influencing cancer progression and metastasis [44].

Our findings indicate that the combined effect of the “dark genes” and their first-order neighboring DEGs regulates cell cycle progression near the critical transition via the JAK–STAT pathway (Fig 4D). The DNB gene *GHR* (growth hormone receptor), identified as the most upstream signaling molecule, does not exhibit a notable alteration in gene expression and is classified as a “dark gene” from a DNFE standpoint [5]. Suppressors of cytokine signaling (*SOCS*) genes inhibit the activation of *STAT3* mediated by *GHR* [45]. The first-order neighboring DEG, *STAT3*, shows obvious changes before and after the tipping point, which may cause dimerization of STAT, which in turn enters the nucleus and directly regulates the expression of downstream signaling genes [46]. Additionally, protein inhibitors of activated STATs have been detected as antagonists of cytokine-regulated signaling, inhibiting the activity of STAT TFs [47]. Moreover, our results indicate that expression of the *CCND2* gene changes notably after entering the critical state, which may facilitate cell cycle progression in cancer cells [48]. These observations support our hypothesis that signals for cell cycle progression are conveyed in a sequential manner across the cancer development process via the JAK–STAT pathway, with the ultimate effects becoming apparent only after the transition at the tipping point.

### Detection of critical states for KIRP

To verify the computational efficacy of our developed method, we applied the DNFE method to two tumor datasets (KIRP, BLCA) from the TCGA database. The applications of the DNFE method to KIRP are depicted in the main text, and the results of the BLCA study are presented in S4 File and S4 Fig.

**Fig 5:**
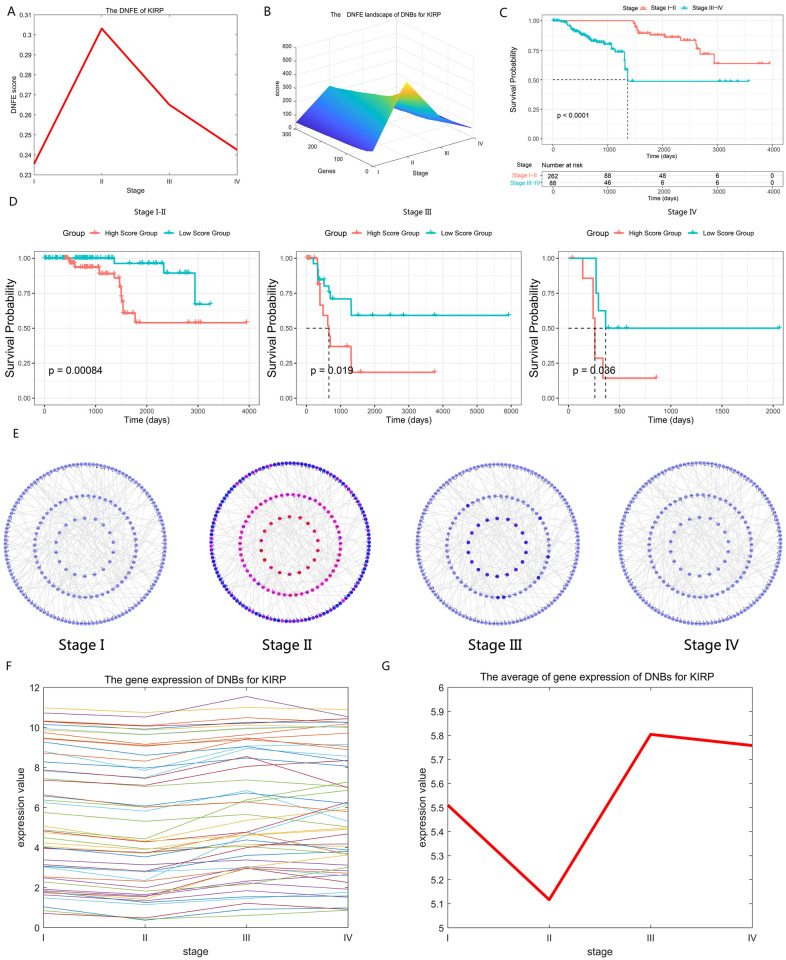
Detection of critical state for kidney renal papillary cell carcinoma (KIRP). (A)The directed network flow entropy (DNFE) score trajectory for KIRP reveals a notable increase at Stage II, signifying a critical transition point in the disease’s progression. (B) The DNFE landscape for KIRP’s dynamic network biomarkers (DNBs) exhibits a marked increase at Stage II, underscoring the importance of this phase as a critical state in the disease trajectory. (C) A comparison of survival curves for KIRP delineates a stark difference, with patients experiencing an obviously longer survival time prior to Stage II compared to those post-Stage II, highlighting the prognostic significance of this stage. (D) The survival curves for KIRP, stratified by high-score and low-score samples, are contrasted to demonstrate the impact of DNFE scores on patient outcomes. High-score samples (red curves) suggest a poorer prognosis, while low-score samples (light blue curves) indicate a more favorable survival rate. (E) The dynamic development of the DNB directed network structure for KIRP indicates that early-warning signals before critical transitions can be identified at Stage II. (F) The gene expression analysis of DNBs is presented. (G) In KIRP, the average gene expression of DNBs fails to differentiate the tipping point from other points; however, the DNFE method has the capacity to accurately identify the tipping points.

Fig 5A reveals a sudden increase in DNFE scores at Stage II, indicating the tipping point. This is followed by the distant metastasis of cancer, leading to an obvious deterioration in the patient’s tumor state. The top 5% of genes with the highest DNFE scores at the tipping point were detected as DNBs. Fig 5B illustrates the tipping point, where the DNFE score in DNBs shows an evident increase during Stage II. Cancer-related mortality is largely attributed to tumor progression, including metastasis, which entails the migration of carcinoma cells from the primary tumor to distant sites [49]. Consequently, identifying the critical state is essential for preventing or preparing for potential deterioration, allowing for timely and effective clinical interventions. A prognostic analysis was conducted using clinical data from KIRP samples, focusing on durations leading up to and subsequent to the critical state (Stage II). Fig 5C illustrates a notable difference in the survival curves for KIRP samples prior to and following Stage II. Additionally, the survival times of samples preceding the tipping point are considerably longer compared to those succeeding it. The abrupt decline in survival times among patients intensely indicates that Stage II represents the tipping point in KIRP. The development of KIRP is a multistage process, with the risk of deterioration increasing from one stage to the next. We further investigated the deterioration risk of KIRP by categorizing samples from each stage into two categories according to the median DNFE score: high-score samples and low-score samples. Subsequently, we conducted a comparison of the prognoses of these two categories. Fig 5D illustrates that the red curve denotes the survival probability of the high-score samples, while the light blue curve denotes the survival probability of the low-score samples. Statistically, the p-values obtained from the KIRP prognostic analysis were 0.00084 (Stage I-II), 0.019 (Stage III), and 0.036 (Stage IV). The p-values resulting from the log-rank test in the Kaplan–Meier analysis are below 0.05, indicating that the prognostic analysis is significant at a statistical level. Individuals within the high-score cohort at Stages I–II exhibit a longer survival duration than their counterparts in the low-score cohort. Conversely, in the post-critical state, the high-score group experiences a markedly reduced survival duration when juxtaposed with the low-score group. This post-critical state high-score group is characterized by an elevated risk of disease progression and an unfavorable prognosis. Fig 5E delineates the DNB’s molecular network, which is modulated by specific genes, revealing a distinct architecture for Stage II as opposed to other stages. Collectively, these findings underscore the pivotal nature of Stage II as a critical juncture in the disease trajectory. Furthermore, as shown in Fig 5F and 5G, utilizing DNFE scores derived from DNB genes, we were able to delineate the critical state from other stages with precision (Fig 5A), while merely using the gene expression levels of DNB genes fails.

### Revealing functional roles of DNBs and “dark genes” in the progression of KIRP

**Fig 6:**
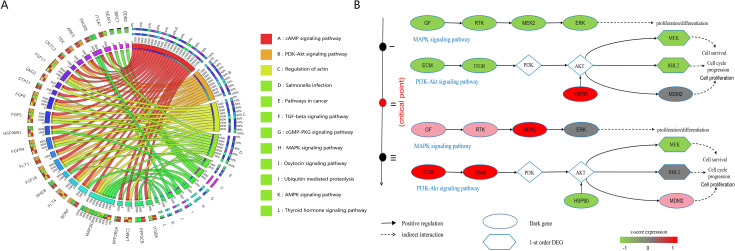
Directed network flow entropy (DNFE) signal biomarkers are involved in important biological processes for kidney renal papillary cell carcinoma (KIRP). (A) Dynamic network biomarkers (DNBs) in KIRP are integral to various biological processes and are implicated in key Kyoto Encyclopedia of Genes and Genomes pathways. (B) Transition dynamics of differentially expressed genes, particularly those before and after the critical state, are influenced by potential “dark genes”. “Dark genes” are a unique class of genes that, despite not showing differential expression at the traditional significance threshold (P<0.05; t-test), exhibit an evident change in their DNFE values (P<0.05).

Moreover, the enrichment analysis of DNBs has substantiated their pivotal role in the course of KIRP progression (Fig 6A). At the critical state, these biomarkers are notably enriched in signaling pathways that are intricately linked to the pathogenesis of the disease. Specifically, DNBs are found to be concentrated in pathways, such as the PI3K–Akt signaling pathway, the MAPK signaling pathway, etc. Fig 6B illustrates the fundamental mechanism elucidated through the functional analysis of “dark genes” and their first-order neighbors. It should be noted that there exists a signaling cascade responsive to “dark genes” in both the MAPK and PI3K/Akt signaling pathways that is crucial for cell proliferation. In the MAPK signaling pathway, the recognized “dark gene” *MAPK9* serves as a pivotal gene within the c-Jun N-terminal kinase subclass pathway, potentially triggering various upstream signals that promote cell reproduction and differentiation [50].

In the PIK3/Akt signaling pathway, an increase in *IGF1R*, *SOS1*, and *SOS2* levels leads to the activation of *RAS* and subsequently activates *RPS6KA3*, for which the cascade may contribute to mitogenic effects [51]. The downstream responses triggered by “dark genes” are closely linked to cell reproduction and differentiation processes. The persistent expression of relevant genes within these pathways from Stage I through Stage III is crucial for enhancing both proliferation and differentiation. This aligns with the literature findings [31] suggesting that the identified tipping point could serve as an important milestone in directing pluripotent stem cells towards the definite endoderm.

### Predicating prognostic effects of “dark genes” for KIRP

**Fig 7:**
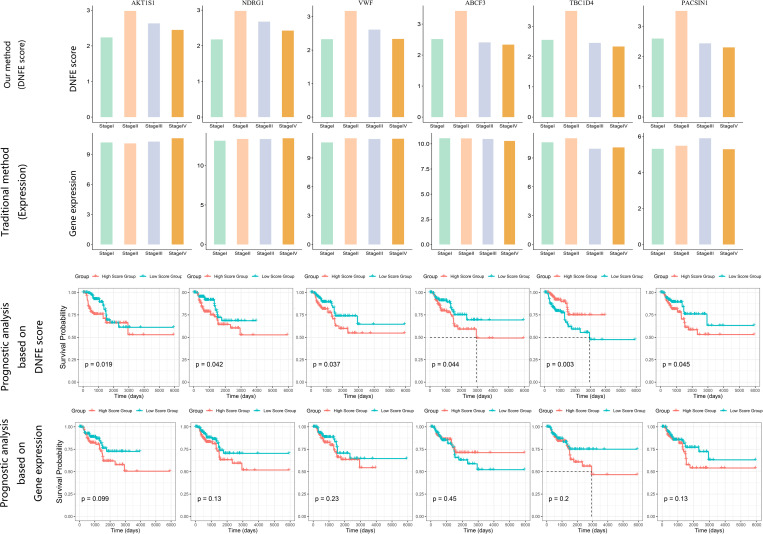
Prognostic comparative analysis between gene expression and directed network flow entropy (DNFE) scores of six “dark genes” for kidney renal papillary cell carcinoma.

As shown in Fig 7, *AKT1S1, NDRG1*, *VWF*, *ABCF3*, *TBC1D4*, and *PACSIN1* have been identified as “dark genes”. Upon reaching the tipping point, the DNFE scores of “dark genes” exhibit heightened sensitivity and evident upward trends leading up to the critical state of tumor deterioration, as opposed to the gene expression data. This trend is more evident than the changes observed in gene expression data. Furthermore, “dark genes” have been shown to be crucial for cancer prognoses. To assess the prognostic value of “dark genes”, we categorized all samples into high-score group and low-score group, which are established based on gene expression and DNFE scores. Fig 7 illustrates that for the “dark gene” *VWF*, there is a noticeable elevation in the DNFE score at the tipping point of KIRP, in contrast to the relatively stable gene expression. Furthermore, a stratified analysis of the DNFE score and the gene expression of the “dark gene” *VWF* revealed prognostic significances of 0.037 and 0.23, respectively. Statistically, this indicates that the subgroups defined by the DNFE score can effectively segregate KIRP patients into groups with varying survival outcomes, a distinction that gene expression alone fails to achieve, underscoring the DNFE score’s utility in the early diagnosis of diseases. Similar findings were observed for other “dark genes”, further validating the DNFE score’s efficacy in disease prognosis.

Moreover, these “dark genes” hold a key position in the progression of cancer. For example, the downregulated expression of *VWF* in tumor tissues is related to *ERS* model genes, which implies that it may have a potential essential function in cancer progression [52]. The EGFR-mediated signaling pathways through *PTPN11* (*SHP2*) and *AKT1S1 (PRAS40*), along with the enhanced anti-apoptotic signaling pathways resulting from acquired resistance to cetuximab, provide multiple opportunities for identifying and validating biomarkers, signaling pathways, and resistance mechanisms for clinical improvements in cancer treatment [53]. Cell proliferation assays, colony formation assays, and cell migration experiments indicate that overexpressing circRNA-*TBC1D4* aids in the migration of neuroblastoma (NB) cells but does not enhance in vitro proliferation or colony formation. These results suggest that circRNA-*TBC1D4* is generally downregulated in NB cells and may serve as a negative biomarker for this type of cancer [54].

Comparative analysis of prognostic outcomes based on gene expression and DNFE scores for the “dark genes” *AKT1S1*, *NDRG1*, *VWF*, *ABCF3*, *TBC1D4*, and *PACSIN1* revealed that DNFE scores have greater sensitivity to tipping points before disease deterioration and provide superior prognostic predictions compared to gene expression levels. The prognosis associated with these “dark genes” indicates apparent differences in survival times between the two sample groups—those with high DNFE scores and those with low DNFE scores—highlighting the greater relevance of DNFE scores over gene expression in predicting outcomes.

### Detecting changes in immune signatures for SLE-NC

DNFE not only predicts the critical state but also accurately detects changes in immune signatures in vivo. To demonstrate the efficiency of the proposed method based on a body fluid dataset, we utilized the DNFE method with a blood dataset of patients with a non-complicated SLE pregnancy (SLE-NC).

**Fig 8:**
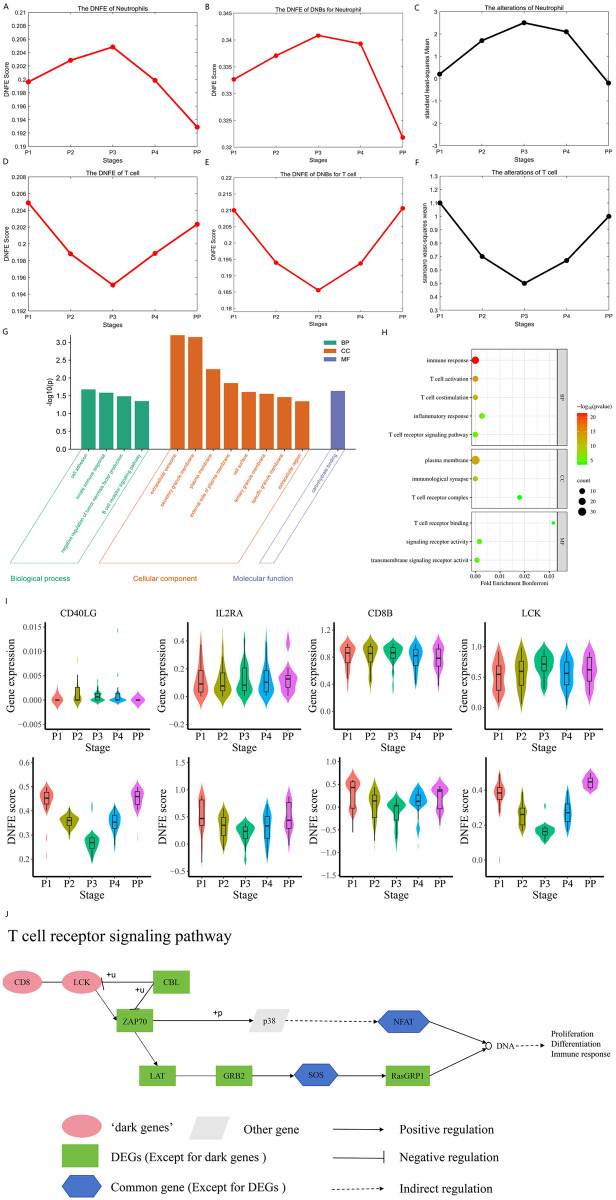
Identification of critical states and functional roles revealed by dynamic network biomarkers (DNBs) and non-differential “dark genes” for SLE-NC. (A) The directed network flow entropy (DNFE) score curve for neutrophils. (B) The DNFE score curve of DNBs for neutrophils. (C) The alterations curve for neutrophils in the original paper. (D) The DNFE score curve for T cells. (E) The DNFE score curve of DNBs for T cells. (F) The alterations curve for T cells in the original paper. (G) A bar plot representing enriched gene ontology (GO) terms, with names and IDs indicated, sorted by the number of associated genes. (H) Dot plot for the enriched GO terms. (I) The comparison of changes in immune signatures in vivo between gene expression and DNFE scores of the “dark genes” *CD40LG*, *IL2RA*, *CD8B*, and *LCK*, for which DNFE scores are more sensitive to changes compared with gene expression data. (J) The regulation of related marker genes in the T cell receptor signaling pathway.

For neutrophils, the DNFE score for marker genes (Fig 8A) and the DNFE score for DNBs (Fig 8B) both increased from P1, reaching a maximum at P3, and subsequently decreased below P1 levels at PP, which is consistent with the alterations observed in neutrophils reported in the original manuscript (Fig 8C). For T cells, the DNFE score in marker genes (Fig 8D) and the DNFE score in DNBs (Fig 8E) decreased from P1, reached the minimum at P3, and subsequently increased below P1 levels at PP, which is consistent with the alterations observed in T cells reported in the original manuscript (Fig 8F). Immunological changes in neutrophils and T cells at five time points were detected by applying the algorithm to the SLE-NC data, which aligns with the transcriptional changes surrounding embryo implantation monitored during assisted reproductive technology, thereby demonstrating the efficacy of DNFE.

### Revealing functional roles of DNBs and “dark genes” for SLE-NC

As shown in Fig 8A and 8C, the neutrophil cell signature steadily increased from P1, reaching the highest levels at P3, and decreased below the P1 baseline from P3 after delivery. In the early stages of pregnancy, the increase in estrogen and progesterone may stimulate the production and release of neutrophils in the bone marrow, resulting in an increase in the number of neutrophils. This helps to enhance the immune system’s response capability to protect the mother and the fetus from the risk of infection. In the later stages of pregnancy, as the fetus develops and the mother’s immune system gradually recovers, there may be a decrease in inflammation levels and a balance adjustment in the immune system, leading to a decrease in the number of neutrophils. This adjustment may help to prevent excessive immune responses while maintaining the immune system in an appropriate functional state to sustain the health of the mother and the fetus [8]. This result supports the notion that the regulatory role of the major transcriptional networks during healthy pregnancies is conserved in SLE pregnancies [55]. The T cell signature steadily decreased from P1, reaching the lowest levels at P3, and increased above the P1 baseline from P3 after delivery (Fig 8D and 8F). In the early stages of pregnancy, the number of T cells decreases to prevent the mother from having an immune response to the fetus. As pregnancy progresses, the fetus develops its own immune system, and the mother’s immune system gradually becomes more active, leading to an increase in T cell numbers to protect both the mother and the fetus from the risk of infection [56]. The findings suggest that the modulation of this pathway is essential for achieving successful pregnancies in SLE [55]. Immunological changes in neutrophils and T cells at five time points were detected by applying the algorithm to the SLE-NC data, which is not only consistent with the transcriptional alterations observed in the vicinity of embryo implantation monitored using assisted reproductive technology but also illustrates the changes in cells from a biological perspective, demonstrating the efficacy of DNFE.

Subsequently, we conducted GO enrichment analysis on the marker genes that may hold important biological relevance in SLE-NC. Some genes were found to be enriched in biological processes related to immunity or defense, e.g., the “innate immune response” (GO:0045087), “B cell receptor signaling pathway” (GO:0050853), “immune response” (GO:0006955), and others in the Gene Ontology analysis (Fig 8G and 8H). The onset of SLE is impacted by genetic susceptibility and dysregulation of the immune response [57]. The pathway involving T cell receptor signaling is vital for the immune system, regulating the activation, proliferation, and function of T cells. For pregnant women with SLE, the balance of the immune system is more fragile, which may lead to exacerbated autoimmune reactions and disease exacerbation. The abnormal activation or dysregulation of the T cell receptor signaling pathway is likely to be associated with the development and clinical manifestations of SLE during pregnancy.

To delve deeper into the elusive elements within the regulatory mechanisms of SLE-associated pathways, we conducted a comparative analysis between the DNB genes and DEGs. The investigation revealed the presence of certain genes within the DNBs that, while not exhibiting differential expression at the molecular level, have a notably high DNFE score at the network level. These genes, which elude traditional detection methods, are referred to as “dark genes”. For T cells, as shown in Fig 8I, *CD40LG*, *IL2RA*, *CD8B*, and *LCK* were found to be “dark genes”. DNFE scores reflect the trends in T cells better than those based on gene expression data.

To provide greater clarity on the pathway associations of T cell genes, we concentrated on analyzing the KEGG pathways related to the T cell receptor signaling pathway that are most relevant to SLE progression (Fig 8J). *CD8*, identified as a “dark gene”, is implicated in the T cell receptor signaling pathway, which was a key regulator. *CD8* contributes apparently to the immune response, T cell activation, and regulation of the immune response, etc. The elevated levels of *ICOS* and *CXCR5* on *CD8* T cells from SLE patients suggest a pathogenic *CD8* T cell subset exhibiting amplified cytotoxicity and potentially facilitating a stronger antibody response through B cell activation [58].

## Discussion

The ability to discern pivotal transitional phases within biological phenomena, such as recognition of the prodromal phase preceding tumorigenesis and the critical junctures of cell lineage specification that occur during embryogenesis, holds great significance. The recognition of indicators that precede the onset of pathological conditions is vital for timely interventions. However, the characterization of biological system dynamics and accurate detection of tipping points or critical states from datasets is challenging due to the resemblance between pre-transition states and critical states concerning the phenotype and average gene expression. In this paper, we introduce an innovative approach that utilizes directed network analysis to identify early-warning signals in biological processes, marking a departure from conventional techniques that focus solely on differential gene expression data. This method leverages the topological properties of networks to uncover subtle yet obvious changes that may precede critical transitions in biological systems. Our results indicate that the proposed DNFE method can achieve effective results in detecting the critical states of six real datasets, identifying DNBs, and revealing the intrinsic molecular mechanisms occurring during the progression of the disease. This method also enhances our understanding of the regulatory relationships between genes. Additionally, we found that “dark genes” from three embryonic differentiation datasets, two cancer datasets, and a blood dataset have an important influence on the progression of biological processes or pathways. Numerical simulations demonstrated the efficiency and resilience of the DNFE method compared to existing approaches, as well as its ability to handle vast amounts of data or large-scale datasets.

The methods [59,60] of criticality and tipping points used pseudotime analysis to simulate the trajectory of cell differentiation. While this approach achieves a continuous control variable, it does not provide actual time points. As a result, they could only make assumptions and use the pseudotime to trace the developmental trajectories of cells. In contrast, the datasets in our manuscript are associated with specific time points or stages, which are discrete-time data, including both time-series data and stage-course cancer data spanning multiple years. Based on dynamic system theory, a biological process—whether characterized by discrete-time data or continuous control variable data—can be described as a dynamical system. For discrete-time data, we employ difference equation models, whereas for continuous control variable data, we utilize differential equation models. And a tipping point is mathematically defined as a bifurcation point of the corresponding dynamical system. A tipping point corresponds a critical state, after which the system state shifts abruptly from one stable state to another attraction including another stable state or periodic oscillation.

In conclusion, we introduce an effective and robust computational method, DNFE, that is competent in identifying critical states from bulk and single-cell data and identifying corresponding DNBs. The DNFE method also shows promising potential for exploring potential molecular mechanisms of disease progression and discovering new network biomarkers and “dark genes”.

DNFE is a model-free and data-driven method. Nevertheless, DNFE also has limitations. To begin, every phase requires an adequate number of samples: a meager sample size can skew the method’s accuracy, while an excessively large sample size can dampen the computational efficacy and waste computational resources. Furthermore, while we primarily corroborated our analytical findings through comparisons with the literature and subsequent analyses, experimental confirmation was not sufficiently addressed in our work and remains an essential next step. TFs, for example, are crucial for understanding cellular functions, developmental biology, and the underpinnings of diseases. We forecasted TFs by pinpointing the “dark genes” with the aid of web-based instruments in this research. Nevertheless, this technique might not be completely precise, and further experimental corroboration is warranted.

## Methods

### Dataset

DNFE is utilized across six real datasets, including three single-cell expression datasets, a blood dataset, and two tumor datasets. mESC-to-MP, hESC-to-DEC, MEF-to-neuron, and SLE-NC data in this study are downloaded from the NCBI Gene Expression Omnibus (GEO) database (NCBI GEO: GSE79578 for mESC-to-MP data, GSE75748 for hESC-to-DEC data, GSE67310 for MEF-to-neuron data, and GSE108497 for SLE-NC data), which are publicly accessible at http://www.ncbi.nlm.nih.gov/geo. KIRP and BLCA data in this study are downloaded from TCGA database and are publicly accessible at http://cancergenome.nih.gov.

### Data preprocessing and functional analysis

We analyzed the preprocessed gene expression datasets that were measured for SLE-NC. The datasets were obtained from the NCBI GEO database (access ID: GSE108497) and were not raw data offered by Hong et al. [21]. The datasets encompassed 31,621 probes.

Afterward, we processed the downloaded matrices to create the necessary gene expression matrices (GEMs) by converting IDs and removing duplicates and null values. When multiple probes were associated with a single gene, their values were averaged to yield GEMs containing 19,404 genes.

DNFE excels at forecasting critical states and precisely identifying in vivo shifts in immune signatures. Our analysis encompassed 512 samples in total. We utilized samples collected at designated gestational time frames (P1: < 16 weeks gestation [WG]; P2: 16–23 WG; P3: 24–31 WG; P4: 32–40 WG; and 8–20 weeks postpartum [PP]) for the microarray examinations. Pregnant patients with SLE were categorized based on the pregnancy outcomes: those without complications (NC), those with preeclampsia (PE), and those with other fetal-related complications without the presence of PE (OC). We performed immune signal probing assays based on SLE-NC data.

The enrichment analysis of DNBs is based on the Gene Ontology Consortium (http://geneontol-ogy.org) and DAVID Bioinformatics Resources (https://david.ncifcrf.gov/). The TFs are predicted through CHEA3 (https://maayanlab.cloud/chea3/). Protein–protein interaction (PPI) networks are drawn based on STRING (https://string-db.org/) and the client software Cytoscape (https://cytoscape.org/).

### Outline of the DNFE algorithm

**Fig 9:**
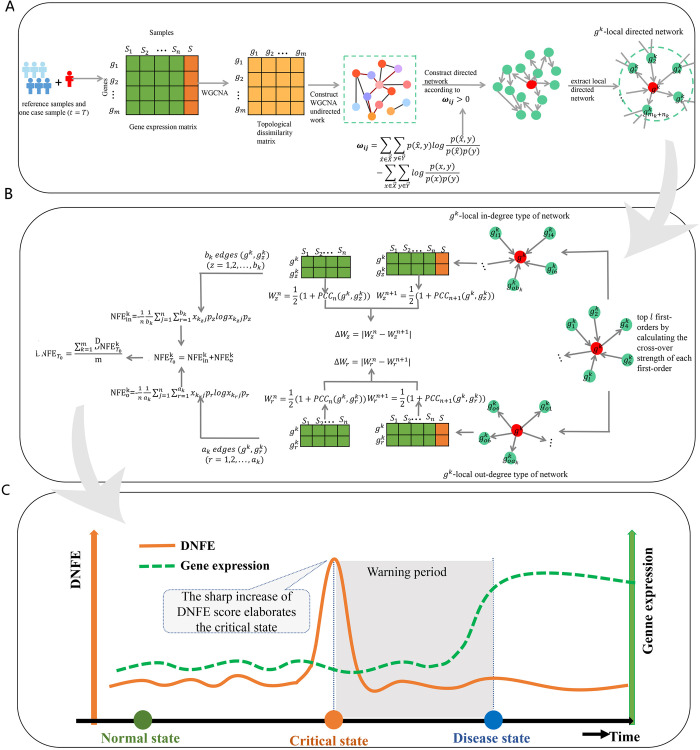
Schematic of the directed network flow entropy (DNFE) algorithm. (A) Given a set of control cells or samples and a corresponding set of case cells/samples at point T, we generated the specific directed network using rewire weighted gene co-expression network analysis with the direction determination index. (B) The global DNFE is calculated to pinpoint the tipping points of complex biological processes at point T. (C) In the dynamic biological progression, the DNFE score is at a low level as the system is in a normal state, but it surges dramatically as the system moves toward the critical state. This sudden rise in the DNFE score signifies the tipping point of the biological process.

Given a collection of control cells/samples and a corresponding set of case cells/samples, we constructed a specific directed network. This construction is based on the rewiring of a weighted gene co-expression network analysis, guided by a direction determination index$\omega_{ij}$ (Fig 9A). Subsequently, the DNFE is calculated and utilized to detect the early warning signals of critical transitions during the development of complex biological processes (Fig 9B). Throughout the dynamic evolution of these processes, the DNFE score remains low while the system is stable. However, it shows a marked increase as the system nears a critical state. This sudden rise in the DNFE score acts as a signal for impending tipping points in the biological process (Fig 9C). The theoretical background of our model can be seen in S1 File.

### Algorithm for disease prediction based on DNFE

DNFE is designed to identify tipping points during the development of complex diseases and identify DNBs. Given a set of reference/control samples that represent a relatively normal state, the subsequent algorithm is formulated to detect the tipping point based on a specific sample. A flow diagram of the DNFE is presented in Fig 1, with detailed procedures outlined in the following subsections.

### Construction of the directed network

The WGCNA network is constructed based on reference samples and case samples. Based on the WGCNA network and gene expression data, the global directed network can be established using a direction determination index, which is defined as follows:


$$\omega_{i,j}=\sum_{\hat{x}\in \vec{\hat{X}}}\sum_{y\in \vec{Y}}p(\hat{x},y)log\frac{p(\hat{x},y)}{p(\hat{x})p(y)}-\sum_{x\in \vec{X}}\sum_{y\in \vec{Y}}p(x,y)log\frac{p(x,y)}{p(x)p(y)}$$ (1)

Here, vectors $\vec{U}$ and $\vec{V}$ represent the expression profiles of genes $g_{i}$ and $g_{j}$ in all samples, respectively; $\vec{\hat{X}}$ and $\vec{X}$ are defined as $\vec{\hat{X}}$ = $(\vec{U}+\vec{V})/\sqrt{2}$, which ensures that information from $\vec{U}$ and $\vec{V}$ has equal weight and prevents the exponential growth of the feature values during the crossover process and improves the numerical stability of the algorithm; $\vec{X}$ = $\vec{U}$, $\vec{Y}$ is the phenotype representing the binary vector for each sample (0─1); $p(\hat{x},y)$ is the joint probability density function (pdf) of $\vec{\hat{X}}$ and $\vec{Y}$; p(x,y) represents the pdf of$\vec{X}$and $\vec{Y}$; and $p(\hat{x})$,p(x),p(y) represent the edge pdf of $\vec{\hat{X}}$, $\vec{X}$, $\vec{Y}$, respectively. The positive determination value indicates that the integration of the gene $g_{j}$ is an improvement in the gene $g_{i}$’s mutual information; that is, there exists a directed edge ($g_{i},g_{j}$) from $g_{i}$ to $g_{j}$ in the directional network. Explicitly, a directed edge ($g_{i},g_{j}$) exists from gene $g_{i}$ to $g_{j}$ when $\omega_{i,j}$ is greater than zero; otherwise, no directed edge ($g_{i},g_{j}$) is present. We constructed the global directed network $N^{global}$ using this approach, where each directed edge ($g_{i},g_{j}$) from gene $g_{i}$ to $g_{j}$ is decided by direction determination index $\omega_{i,j}$.

### Extraction of the gene module from the global network

Each local gene module $M^{k}(k=1,\ldots ,m)$ is extracted from the global network. In addition, in the analysis of gene modules, the scope is limited to the immediate surroundings of each gene, encompassing only its first-order and second-order neighbors. The local gene module $M^{k}$ centers on gene $g^{k}(k=1,2,...,m)$ and $m_{k}$ first-order out-degree neighbors $\left\{g_{o1}^{k},...,g_{om_{k}}^{k}\right\}$, $n_{k}$ first-order in-degree neighbors $\left\{g_{i1}^{k},...,g_{in_{k}}^{k}\right\}$.

### Determination of the local directed network

There are $p_{j}$ second-order out-degree neighbors $\left\{g_{jo1}^{k},...,g_{jop_{j}}^{k}\right\}$ and $q_{j}$ second-order in-degree neighbors $\left\{g_{ji1}^{k},...,g_{jiq_{j}}^{k}\right\}$ for each first-order neighborhood gene $g_{j}^{k}$ (*j*$=1,2,...,m_{k}+n_{k})$. The crossover strength of each first-order neighborhood gene $g_{j}^{k}$ was calculated as follows:


$$D_{j}=\lambda D_{j}^{in}+(1-\lambda {)D}_{j}^{out}$$ (2)

where λis a constant, 0<λ<1. The choice of λ affects the final calculated gene importance ranking or the results of network structure analysis. In the process of determining the optimal value of λ, we alliteratively modified it within our algorithm to observe if the resulting outcomes matched those observed clinically. In this study, the critical thresholds of λ was set to 1/5. The algorithms discussed in this article are implemented under the similarity weight principle, where a higher edge weight correlates with a shorter distance between points, suggesting a closer relationship. Therefore, the in-intensity of the node can reflect the importance of the node. $D_{j}^{in}=\sum_{i=1}^{q_{j}}\omega_{ij}$ is the in-strength of the first-order neighborhood genes, and $D_{j}^{out}=\sum_{i=1}^{p_{j}}\omega_{ij}$ is the out-strength of the first-order neighborhood genes. $\omega_{ij}$ represents the weight of the directed edge$(g_{i},g_{j}$), which is defined as follows:


$$\omega_{ij}=\frac{W_{ij}}{\sum_{i=1}^{p_{j}/q_{j}}W_{ij}}$$ (3)

Where


$$W_{ij}=\frac{\left(n-1)\Delta PCC(g_{i}^{k},g_{j}^{k}\right)}{1-({{PCC}^{n}(g_{i}^{k},g_{j}^{k}))}^{2}}$$ (4)

In this manner, the crossover strength of each first-order neighborhood gene $g_{j}^{k}$ was ranked in descending order, and the top l first-order neighborhood genes were selected with the central gene $g^{k}$ to form a local central network $N^{k}(k=1,2,...,m)$.

### Calculating a local DNFE score for each local directed network

We can obtain the local directed network $N^{k}(k=1,2,...,m)$, and each local directed network $N^{k}$ is concentrated around gene $g^{k}$, including $a_{k}$ first-order out-degree neighbors $\left\{g_{o1}^{k},...,g_{oa_{k}}^{k}\right\}$ and $b_{k}$ first-order in-degree neighbors $\left\{g_{i1}^{k},...,g_{ib_{k}}^{k}\right\}$, and ${a_{k}+b}_{k}$ =l.

For the local directed network (with $a_{k}$ first-order out-degree neighbors and $b_{k}$ first-order in-degree neighbors) centered on gene $g^{k}$, its relevant local DNFE score at t=T is characterized below:


$${DNFE}_{T}^{k}={NFE}_{o}^{k}+{NFE}_{in}^{k}$$ (5)

where ${NFE}_{o}^{k}$ and ${NFE}_{in}^{k}$ are described below:


$${NFE}_{o}^{k}=-\frac{1}{n}\frac{1}{a_{k}}\sum_{j=1}^{n}\sum_{r=1}^{a_{k}}x_{k_{r}j}p_{r}\log x_{k_{r}j}p_{r}$$ (6)

With


$$P_{r}=\frac{{\Delta W}_{r}}{\sum_{r=1}^{a_{k}}{\Delta W}_{r}}$$ (7)

$${\Delta W}_{r}=|W_{r}^{n}-W_{r}^{n+1}|$$ (8)

$$W_{r}=\frac{1}{2}(1+{PCC}_{out}(g^{k},g_{r}^{k}))$$ (9)

and


$$\begin{array}{c} {NFE}_{in}^{k}=-\frac{1}{n}\frac{1}{b_{k}}\sum {\mathrm{\backslash nolimits}}_{j=1}^{n}\sum {\mathrm{\backslash nolimits}}_{z=1}^{b_{k}}x_{k_{z}j}p_{z}\log x_{k_{z}j}p_{z} \end{array}$$ (10)

With


$$P_{z}=\frac{{\Delta W}_{z}}{\sum_{z=1}^{b_{k}}{\Delta W}_{z}}$$ (11)

$${\Delta W}_{z}=|W_{z}^{n}-W_{z}^{n+1}|$$ (12)

$$W_{z}=\frac{1}{2}(1+{PCC}_{in}(g^{k},g_{z}^{k}))$$ (13)

where $W^{n}{andW}^{n+1}$ are the transformed Pearson correlation coefficients (PCCs) of gene expression regarding the center gene $g^{k}$ and its first-order neighbor $g_{j}^{k}$ using n reference samples and n+1 mixed samples (composed of reference samples and one specific sample), respectively. $x_{k_{r}j}$ and $x_{k_{z}j}$ are the expression data of gene $g_{r}^{k}$ and $g_{z}^{k}$ in sample j.

### Calculation of the DNFE score of the global directed network

At time point t=T, the DNFE score for the perturbed sample is defined as follows:


$${DNFE}_{T}=\frac{\sum_{k=1}^{m}{{DNFE}_{T}}^{k}}{m}\mathrm{\backslash ]}$$ (14)

The DNFE score, assessed at successive time points, serves as a metric of the overarching disruption induced by the case samples within the biological network. We repeat previous steps to calculate the other score at the different time t=T. If ${DNFE}_{T}$ sharply increases, then T is the tipping point, and the top 5% genes in terms of ${DNFE}_{T}$ are DNBs in this work. Otherwise, return to Fig 9A by taking next time point (i.e., T + 1) as a candidate critical state. To evaluate the capacity of the CDNFE score in capturing critical state, we applied one-sample t-test ($S=\frac{\sqrt{n}\left(mean(\hat{X})-x\right)}{SD\left(\hat{X}\right)}$), and the time point t can be conceived as critical state if the DNFE score ${DNFE}_{T}$ meets the following two conditions: (i) ${DNFE}_{T}$> ${DNFE}_{T-1}$, and (ii) ${DNFE}_{t}$ is statistically different (P < 0.05) from the prior information. Genes with the top 5% highest scores are classified as DNBs at the critical state. The DNFE algorithm primarily describes the fluctuations within the network, serving as a fundamental tool for quantifying the network’s criticality or tipping point. This approach offers a valid and stable early warning signal before critical transitions.

## Supporting information

S1 File Theoretical background. (PDF)

S2 File Numerical simulation details. (PDF)

S3 File Pinpointing cell fate commitment during the cellular differentiation process. (PDF)

S4 File Identifying the critical transition for bladder cancer. (PDF)

S1 Fig Model of an eleven-molecule network. This illustration of a molecular network features eleven nodes, with their dynamic regulatory interactions defined based on the stochastic system in Eq. (S1) in S1 File. The positive or negative regulatory connections among the nodes are reflected by the edges.
(JPG)

S2 Fig Analytical results for mouse embryonic stem cell (mESC)-to-mesoderm progenitor (MP) cell data. (A) The temporal fluctuations in the directed network flow entropy (DNFE) scores during the mESC-to-MP transition reveal the impending critical state at the 24 h mark. (B) The DNFE landscape for the mESC-to-MP transition demonstrates an apparent surge in the DNFE score at 24 h, underscoring its utility in pinpointing critical developmental stages. (C) The top 20 upstream hub transcription factors exert regulatory control over 89% of the dynamic network biomarker (DNB) genes detected at 24 h, highlighting the pivotal role of these factors in the gene regulatory network. (D) Analysis of Kyoto Encyclopedia of Genes and Genomes pathway enrichment for “dark genes” involved in the mESC-to-MP process. (E) The signaling genes within the PI3K/Akt pathway, which are enriched at the tipping point, exhibit distinct regulatory patterns before and after lymph node metastasis. These genes are instrumental in the progression of cancer.
(JPG)

S3 Fig Analysis results for mouse embryonic fibroblast (MEF)-to-neuron data. (A) The trajectory of the directed network flow entropy (DNFE) score throughout the MEF-to-neuron reprogramming timeline is marked by notable escalation at day 20, signifying a tipping point that heralds a critical phase in the cellular transition. (B) The DNFE landscape of dynamic network biomarkers (DNBs) during the MEF-to-neuron transition reveals a pronounced uptick in the DNFE score at day 20, which is indicative of a tipping point. (C) The temporal dynamics of DNBs throughout the MEF-to-neuron transition are characterized by a discernible pattern of change, with the critical state detectable at day 20. (D) Top 20 upstream hub transcription factors are able to regulate 89% of the identified DNB genes at day 20. (E) The results of the regulatory association between the top 20 transcription factors are shown as a network. (F) The top five transcription factors (TFs) identified using each library, with these TFs represented along the columns and the query genes along the rows. The entries within this matrix are populated based on whether a query gene is present or absent within the target gene set of a library TF at the second tipping point.
(JPG)

S4 Fig Analysis results for bladder cancer (BLCA). (A) The fluctuation in the directed network flow entropy (DNFE) score describes a critical transition at Stage II, suggesting a pivotal moment in the disease’s trajectory. (B) The DNFE landscape analysis delineates the score’s behavior across different stages, providing a visual representation of disease progression. (C) A comparative analysis of survival curves for BLCA reveals a marked disparity in survival times, with an important drop post-stage II. (D) The dynamic development of dynamic network biomarker (DNB) genes is examined, highlighting the changes in these genes’ behavior that coincide with the critical stage. (E) The gene expression of DNBs. (F) The mean gene expression of DNBs is assessed; while it is not sufficient to discern critical states from others, it is complemented by the DNFE method’s ability to detect such stages with greater precision.
(JPG)

S1 Table The comparison between the DNFE algorithm and other GRN methods. (PDF)

S2 Table Detailed hallmark pathway enrichment analysis information. (PDF)
