# Supplementary material for: DNFE: Directed network flow entropy for detecting tipping points during biological processes
Source: PLoS Comput Biol. 2025 Jul 29;21(7):e1013336. doi: 10.1371/journal.pcbi.1013336 (PMC12316398; doi:10.1371/journal.pcbi.1013336)
Supplement: S1 File — (PDF) [file pcbi.1013336.s001.pdf]

Theoretical background

Dynamic Network Biomarkers (DNBs) represent a collection of molecules, such as genes or proteins, or a molecular module that indicates the proximity of a tipping point or critical state, which is often followed by drastic deterioration connected with complex diseases[1]. As a biological or physiological system transitions from a stable normal state towards a critical state, a DNB module or a group of molecules (i.e., variables) emerge and meet the following three statistical conditions:

The standard deviation, representing deviation for each molecule within the module, drastically escalates;

The absolute values of the Pearson correlation coefficients among molecules within the module swiftly rise;

The absolute values of the Pearson correlation coefficients between molecules inside and outside of the modules markedly diminish.

These three conditions form the basis to detect the presence of DNBs or signals indicative of a pre-transition state. Moreover, these criteria are not arbitrary but embody the generic properties of DNB members whenever the system approaches a critical tipping point. These inherent properties have been effectively applied to numerous real diseases for detecting the tipping point or critical state.

The time-specific directed network at a time point T is constructed based on an information-theoretic scheme[2], which provides a direction determination index $\omega_{i,j}$ to evaluate the combined effect of gene combinations over a single gene from the perspective of mutual information (MI).

$$\omega_{ij} = \sum_{\hat{x}\in\vec{\hat{X}}}\sum_{y\in\vec{Y}} p(\hat{x},y) log \frac{p(\hat{x},y)}{p(\hat{x})p(y)} - \sum_{x\in\vec{X}}\sum_{y\in\vec{Y}} p(x,y) log \frac{p(x,y)}{p(x)p(y)}$$

Vectors $\vec{U}$ and $\vec{V}$ represent the expression profiles of genes g_i and g_j in all samples, respectively, $\vec{\hat{X}}$ and $\vec{X}$ are defined as $\vec{\hat{X}}=(\vec{U}+\vec{V})/\sqrt{2}$, $\vec{X}=\vec{U}$, $\vec{Y}$ is the phenotype representing the binary vector for each sample (0-1). $p(\hat{x},y)$ represents the probability density function (pdf) of $\vec{\hat{X}}$ and $\vec{Y}$, and $p(x,y)$ represents the pdf

of $\vec{X}$ and $\vec{Y}$, $p(\hat{x})$,$p(x)$, $p(y)$represent the edge pdf of $\vec{\hat{X}}$, $\vec{X}$, $\vec{Y}$, respectively. The positive determination value indicates that the integration of the gene g_j is an improvement of the gene g_i mutual information (MI), that is, in the directional network, there is a directed edge (g_i, g_j) from g_i to g_j.

A network is fully specified by its adjacency matrix a_{ij} , a symmetric n × n matrix with entries in [0, 1] whose component a_{ij} encodes the network connection strength between nodes i and j. To calculate the adjacency matrix, an intermediate quantity called the co-expression similarity S_{ij} is first defined. The default method defines the co-expression similarity S_{ij} as the absolute value of the correlation coefficient between the profiles of nodes i and j: $s_{ij} = |cor(x_{i,} x_j)|$.The WGCNA package [3] also implements alternative co-expression measures, e.g., more robust measures of correlation (the bi-weight miscorrelation [4] or the Spearman correlation). A signed co-expression measure can be defined to keep track of the sign of the co-expression information. For convenience, we define the co-expression similarity measure such that it takes on values in [0, 1].

Reference

[1] Liu XP, Chang X, Leng SY, et al. Detection for disease tipping points by landscape dynamic network biomarkers. Natl Sci Rev 2019;6(4):775–85.

[2] Sun,D. et al. (2019) Discovering cooperative biomarkers for heterogeneous complex disease diagnoses. Brief. Bioinform., 20, 89–101.

[3] Langfelder P, Horvath S. WGCNA: an R package for weighted correlation network analysis. BMC Bioinformatics 2008; 9: 559.

[4] Wilcox RR. Introduction to Robust Estimation and Hypothesis Testing. Academic Press; 1997.
