# Supplementary material for: DNFE: Directed network flow entropy for detecting tipping points during biological processes
Source: PLoS Comput Biol. 2025 Jul 29;21(7):e1013336. doi: 10.1371/journal.pcbi.1013336 (PMC12316398; doi:10.1371/journal.pcbi.1013336)
Supplement: S2 File — (PDF) [file pcbi.1013336.s002.pdf]

Numerical simulation details

The detection of a pre-disease state using DNFE was conducted through a numerical simulation using a regulatory network composed of eleven genes (**Figure S1**). Such molecular regulatory networks are commonly employed to study various gene regulatory activities, including transcription and translation [1,2], cyclic reactions [3], and non-linear biological processes [4,5,6]. The regulatory interactions amongst the eleven genes in the network are encapsulated in the following eleven differential equations. Within the network, gene regulation adopts a Michaelis-Menten form, with degradation rates linearly proportional to the respective gene concentrations.

$$\frac{dz_1(t)}{dt} = \frac{(10-|p|)z_4(t)}{24(1+z_4(t))} - \frac{10+|p|}{24} z_1(t) + \xi_1(t)$$

$$\frac{dz_2(t)}{dt} = \frac{(6-|p|)z_1(t)}{24(1+z_1(t))} + \frac{(6-|p|)z_4(t)}{24(1+z_4(t))} - \frac{1}{2} z_2(t) + \xi_2(t)$$

$$\frac{dz_3(t)}{dt} = \frac{|p|-8}{12} + \frac{(8-|p|)}{24(1+z_1(t))} + \frac{(8-|p|)}{24(1+z_4(t))} - \frac{2}{3} z_3(t) + \xi_3(t)$$

$$\frac{dz_4(t)}{dt} = \frac{(10-|p|)z_1(t)}{24(1+z_1(t))} - \frac{10+|p|}{24} z_4(t) + \xi_4(t)$$

$$\frac{dz_5(t)}{dt} = -\frac{3}{2} + \frac{z_1(t)}{12(1+z_1(t))} + \frac{2}{3(1+z_2(t))} + \frac{1}{2(1+z_3(t))} + \frac{z_4(t)}{12(1+z_4(t))} + \frac{1}{6(1+z_6(t))} + \frac{1}{6(1+z_7(t))} - \frac{7}{6} z_5(t) + \xi_5(t)$$

$$\frac{dz_6(t)}{dt} = -\frac{1}{2} + \frac{1}{6(1+z_2(t))} + \frac{1}{6(1+z_3(t))} + \frac{1}{6(1+z_5(t))} + \frac{z_7(t)}{6(1+z_7(t))} - \frac{7}{6} z_6(t) + \xi_6(t)$$

$$\frac{dz_7(t)}{dt} = \frac{z_2(t)}{6(1+z_2(t))} + \frac{z_3(t)}{6(1+z_3(t))} + \frac{z_5(t)}{6(1+z_5(t))} + \frac{z_6(t)}{6(1+z_6(t))} - \frac{7}{6} z_7(t) + \xi_7(t)$$

$$\frac{dz_8(t)}{dt} = -\frac{1}{3} + \frac{5z_6(t)}{6(1+z_6(t))} + \frac{5z_7(t)}{6(1+z_7(t))} + \frac{1}{3(1+z_{10}(t))} - \frac{3}{2} z_8(t) + \xi_8(t)$$

$$\frac{dz_9(t)}{dt} = \frac{2z_6(t)}{3(1+z_6(t))} + \frac{2z_7(t)}{3(1+z_7(t))} - \frac{5}{3} z_9(t) + \xi_9(t)$$

$$\frac{dz_{10}(t)}{dt} = \frac{5z_6(t)}{6(1+z_6(t))} + \frac{5z_7(t)}{6(1+z_7(t))} - \frac{11}{6} z_{10}(t) + \xi_{10}(t)$$

$$\frac{dz_{11}(t)}{dt} = \frac{z_6(t)}{1+z_6(t)} + \frac{z_7(t)}{1+z_7(t)} - 2z_{11}(t) + \xi_{11}(t) \qquad (S1)$$

where p is a scalar control parameter and $\varsigma_i(t)(i = 1,2,...,11)$ are Gaussian noises with zero means and covariances $k_{ij} = Cov(\varsigma_i, \varsigma_j)$. $z_i(t)(i = 1,2,...,11)$ represent the concentrations of mRNA-i. In Eq.(S1), there is the degradation rates of

mRNAs $\mathrm{R}=\frac{10+5|p|}{24},\frac{1}{2},\frac{2}{3},\frac{10+5|p|}{24},\frac{7}{6},\frac{7}{6},\frac{7}{6},\frac{3}{2},\frac{5}{3},\frac{11}{6},2$. The stable equilibrium point of the differential equations Eq.(S1) is $\overline{Z}=(\overline{z_1},\overline{z_2},\overline{z_3},\dots,\overline{z_9},\overline{z_{10}},\overline{z_{11}})=(0,0,0,\dots,0)$. The differential equations Eq.(S2) can be transformed into the difference equations $Z(k+1)=f(Z(k),p)$using the Euler scheme [6] with a short time interval Δt. The result is as follows:

$$z_1(k+1)=z_1(k)+[\frac{(10-|p|)z_4(k)}{24(1+z_4(k))}-\frac{10+|p|}{24}z_1(k)+\xi_1(k)]\Delta\mathrm{k}$$

$$z_2(k+1)=z_2(k)+[\frac{(6-|p|)z_1(k)}{24(1+z_1(k))}+\frac{(6-|p|)z_4(k)}{24(1+z_4(k))}-\frac{1}{2}z_2(k)+\xi_2(k)]\Delta\mathrm{k}$$

$$z_3(k+1)=z_3(k)+[\frac{|p|-8}{12}+\frac{(8-|p|)}{24(1+z_1(k))}+\frac{(8-|p|)}{24(1+z_4(k))}-\frac{2}{3}z_3(k)+\xi_3(k)]\Delta\mathrm{k}$$

$$z_4(k+1)=z_4(k)+[\frac{(10-|p|)z_1(k)}{24(1+z_1(k))}-\frac{10+|p|}{24}z_4(k)+\xi_4(k)]\Delta\mathrm{k}$$

$$z_5(k+1)=z_5(k)+[-\frac{3}{2}+\frac{z_1(k)}{12(1+z_1(k))}+\frac{2}{3(1+z_2(k))}+\frac{1}{2(1+z_3(k))}+\frac{z_4(k)}{12(1+z_4(k))}+\frac{1}{6(1+z_6(k))}+\frac{1}{6(1+z_7(k))}-\frac{7}{6}z_5(k)+\xi_5(k)]\Delta\mathrm{k}$$

$$z_6(k+1)=z_6(k)+[-\frac{1}{2}+\frac{1}{6(1+z_2(k))}+\frac{1}{6(1+z_3(k))}+\frac{1}{6(1+z_5(k))}+\frac{z_7(t)}{6(1+z_7(k))}-\frac{7}{6}z_6(k)+\xi_6(k)]\Delta\mathrm{k}$$

$$z_7(k+1)=z_7(k)+[\frac{z_2(k)}{6(1+z_2(k))}+\frac{z_3(k)}{6(1+z_3(k))}+\frac{z_5(k)}{6(1+z_5(k))}+\frac{z_6(k)}{6(1+z_6(k))}-\frac{7}{6}z_7(k)+\xi_7(k)]\Delta\mathrm{k}$$

$$z_8(k+1)=z_8(k)+[-\frac{1}{3}+\frac{5z_6(k)}{6(1+z_6(k))}+\frac{5z_7(k)}{6(1+z_7(k))}+\frac{1}{3(1+z_{10}(k))}-\frac{3}{2}z_8(k)+\xi_8(k)]\Delta\mathrm{k}$$

$$z_9(k+1)=z_9(k)+[\frac{2z_6(k)}{3(1+z_6(k))}+\frac{2z_7(k)}{3(1+z_7(k))}-\frac{5}{3}z_9(k)+\xi_9(k)]\Delta\mathrm{k}$$

$$z_{10}(k+1)=z_{10}(k)+[\frac{5z_6(k)}{6(1+z_6(k))}+\frac{5z_7(k)}{6(1+z_7(k))}-\frac{11}{6}z_{10}(k)+\xi_{10}(k)]\Delta\mathrm{k}$$

$$z_{11}(k+1)=z_{11}(k)+[\frac{z_6(k)}{1+z_6(k)}+\frac{z_7(k)}{1+z_7(k)}-2z_{11}(k)+\xi_{11}(k)]\Delta\mathrm{k} \qquad (S2)$$

It is easy to note that $Z(k)$ is the vector of $Z(t)$ at the time instant $k\Delta t$. The Jacobian matrix of Eq.(S2) can be defined as $J=\left.\frac{\partial f(Z(k);p)}{\partial Z}\right|_{Z=\overline{Z}}$, where

$$J=e^{\Delta t\bullet A} \qquad (S3)$$

With

$$A=\begin{bmatrix}
\frac{-10-|P|}{24} & 0 & 0 & \frac{10-|P|}{24} & 0 & 0 & 0 & 0 & 0 & 0 & 0 \\
\frac{6-|P|}{24} & -\frac{1}{2} & 0 & \frac{6-|P|}{24} & 0 & 0 & 0 & 0 & 0 & 0 & 0 \\
\frac{-8+|P|}{24} & 0 & -\frac{2}{3} & \frac{-8+|P|}{24} & 0 & 0 & 0 & 0 & 0 & 0 & 0 \\
\frac{10-|P|}{24} & 0 & 0 & \frac{-10-|P|}{24} & 0 & 0 & 0 & 0 & 0 & 0 & 0 \\
\frac{1}{12} & -\frac{2}{3} & -\frac{1}{2} & \frac{1}{12} & -\frac{7}{6} & -\frac{1}{6} & -\frac{1}{6} & 0 & 0 & 0 & 0 \\
0 & -\frac{1}{6} & -\frac{1}{6} & 0 & -\frac{1}{6} & -\frac{7}{6} & \frac{1}{6} & 0 & 0 & 0 & 0 \\
0 & \frac{1}{6} & \frac{1}{6} & 0 & \frac{1}{6} & \frac{1}{6} & -\frac{7}{6} & 0 & 0 & 0 & 0 \\
0 & 0 & 0 & 0 & 0 & \frac{5}{6} & \frac{5}{6} & -\frac{3}{2} & 0 & -\frac{1}{3} & 0 \\
0 & 0 & 0 & 0 & 0 & \frac{2}{3} & \frac{2}{3} & 0 & -\frac{5}{3} & 0 & 0 \\
0 & 0 & 0 & 0 & 0 & \frac{5}{6} & \frac{5}{6} & 0 & 0 & -\frac{11}{6} & 0 \\
0 & 0 & 0 & 0 & 0 & 1 & 1 & 0 & 0 & 0 & -2
\end{bmatrix}$$

From Eq.(S3), by taking $\Delta t = 1$, we can obtain eight distinct eigenvalues ($0.92^{|p|}$, 0.61, 0.51, 0.43, 0.37, 0.31, 0.26, 0.22, 0.19, 0.16, 0.14) by taking $\Delta t = 1$. It is obvious that the largest eigenvalue $0.92^{|p|} \to 1$ when $p \to 0$. Therefore, the equilibrium point $\overline{Z}$ is stable when $p \in (0,1]$ and $p = 0$ is a bifurcation point, at which the system undergoes a critical transition. We aimed to detect early-warning signals that indicate the critical transition as a control parameter p approaches a bifurcation point 0. According to DNFE method, for each simulation trial, we used the 28 samples generated when the control parameter p was far away from the bifurcation point $p = 0$ (e.g., $p \in [-0.50, 0.23]$) as the reference samples. Then in each simulation trial, based on the single sample $\{z_1, z_2, \dots, z_{28}\}$ derived for each parameter value $p \in [-0.5, 0.25]$, the DNFE score was calculated following the DNFE method (see Method section in the main text), as shown in Fig 2 in the main

text.

Reference

[1]Khanin R, Vinciotti V, Mersinias V, et al. Statistical Reconstruction of Transcription Factor Activity Using Michaelis–Menten Kinetics. Biometrics 2007; 63:816-823.

[2]Ronen M, Rosenberg R, Shraiman B, et al. Assigning numbers to the arrows: parameterizing a gene regulation network by using accurate expression kinetics. Proc Natl Acad Sci U S A 2002; 99(16):10555-10560.

[3]Sueyoshi C, Naka T. Stability Analysis for the Cellular Signaling Systems Composed of Two Phosphorylation-Dephosphorylation Cyclic Reactions. Computational Molecular Bioscience 2017; 7:33-45.

[4]Chen L, Wang R, Li C, et al. Modeling Biomolecular Networks in Cells: Structures and Dynamics. Springer London 2010.

[5]Chen L, Wang R, Zhang X. Biomolecular Networks: Methods and Applications in Systems Biology. John Wiley & Sons, Inc 2009.

[6]Chen P, Li Y, Liu X, et al. Detecting the tipping points in a three-state model of complex diseases by temporal differential networks. J Transl Med 2017; 15(1):217.
