# Supplementary material for: DNFE: Directed network flow entropy for detecting tipping points during biological processes
Source: PLoS Comput Biol. 2025 Jul 29;21(7):e1013336. doi: 10.1371/journal.pcbi.1013336 (PMC12316398; doi:10.1371/journal.pcbi.1013336)
Supplement: S3 File — (PDF) [file pcbi.1013336.s003.pdf]

# Pinpointing cell fate commitment during the cellular differentiation process

## The results for mESC-to-MP

*Critical states for mESC-to-MP*

As illustrated in Figure **S2A**, sudden increments in DNFE scores at the 24-hour stage depict the identifiable critical state preceding disease deterioration. Figure S2B further demonstrates a sharp rise in DNFE scores at Stage II, strongly suggesting an imminent critical transition.

*Transcription factors in endodermal differentiation of mESCs*

Our research aimed to identify the potential upstream transcriptional regulators of the DNFE genes at the tipping point. We particularly focused on transcription factors (TFs) due to their vital role in defining cell identity and driving cell-fate transitions. Utilizing Ingenuity Pathway Analysis (IPA), we predicted the TFs of DNFE factors, which are designated as DNFE factors. As a result, twenty TFs/DNFE factors were identified relating to the tipping point of 24 hours. This group of transcription factors could potentially regulate 89% of each tipping point's signaling genes, as shown in Figure S2C.

*The underlying signaling mechanisms revealed by DNBs*

Enrichment results revealed that the DNFE signaling genes were primarily concentrated in areas such as the cell cycle, MAPK signaling pathway, PI3K-Akt signaling pathway, and other pathways associated with cancer (Figure S2D). Figure S2E demonstrates that the underlying signaling mechanism of the PI3K/AKT pathway was unveiled through the functional analysis of signaling genes and their first-order differentially expressed gene neighbors. Nominally, upstream growth factor signaling genes like CD19 and PI3K, experiencing a sharp rise in expression after crossing the critical point, suggest the importance of this period for the initiation of these signals. Furthermore, one CSG, AKT, promotes cell cycle maintenance and cell survival by suppressing the expression of FOXO transcription factors[1]. Our study indicates a significant upregulation of CCND1 gene expression post the critical point, potentially

promoting the progression of the cell cycle in cancer cells[2]. Moreover, genes such as FasL, closely associated with cell survival, demonstrate amplified expression prior to the critical phase. These genes, believed to relate directly to apoptotic function[3], suggest that cancer cell opposition to apoptosis is accomplished before the critical state, especially prior to tissue infiltration or lymph node metastasis. These observations align with our hypothesis that cell cycle progression signals are sequentially transmitted throughout the cancer period via the JAK-STAT pathway, with the ultimate effect visible only post the critical point transition.

**The results for MEF-to-neuron**

*Critical states for MEF-to-neuron*

A significant escalation in the DNFE score was observed at day 20 (**Figure S3A**), indicating a critical state or imminent critical transition. Figure S3B demonstrates this critical state at the molecular level, marking a substantial increase in the DNFE score within DNBs at day 20. Throughout the global network, DNBs exhibit particular sensitivity to the critical state. As portrayed in Figure S3C, a DNB molecular directed network, regulated by genes, displays distinct structural differences at day 20 compared to other stages, forecasting upcoming critical states.

*Transcription factors in endodermal differentiation of hESCs*

Our goal was also to discern any potential upstream transcriptional regulators of the tipping point's signaling genes. We estimated the Transcription Factors (TFs) of the signaling factors, and identified twenty TFs/DNFE factors specifically related to the day 20 tipping point. This collection of transcription factors appears to regulate 65% of the signaling genes associated with the tipping point, as shown in Figure S3D. Figure S3E presents a local network, highlighting the mutual regulation between the predicted top 20 transcription factors within the regulatory network. In Figure S3F, heatmap illustrates the result whether the gene appears within the target gene set of the library TF. The top five TFs are included in each library, and each column represent each TFs. C1QBP appeared most frequently in the target gene set of the library TF. overexpression of C1QBP protected the cells against staurosporine-induce

apoptosis, increased proliferation, decreased cellular ATP, and increased cell migration in a wound-healing assay[4].

## Reference

[1]Gan,L. et al. (2009) Cyclin D1 promotes anchorage-independent cell survival by inhibiting FOXO-mediated anoikis. Cell Death Differ., 16, 1408–1417.

[2]Mahdiannasser, Mojdeh et al. “Illuminating the role of lncRNAs ROR and MALAT1 in cancer stemness state of anaplastic thyroid cancer: An exploratory study.” Non-coding RNA research vol. 8,3 451-458. 26 May. 2023.

[3]Ma, Chi-Jiao et al. “Stem Cell Therapies for Intervertebral Disc Degeneration: Immune Privilege Reinforcement by Fas/FasL Regulating Machinery.” Current stem cell research & therapy vol. 10,4 (2015): 285-95.

[4]McGee, Allison M et al. “The mitochondrial protein C1qbp promotes cell proliferation, migration and resistance to cell death.” Cell cycle (Georgetown, Tex.) vol. 10,23 (2011): 4119-27.
