# Supplementary material for: DNFE: Directed network flow entropy for detecting tipping points during biological processes
Source: PLoS Comput Biol. 2025 Jul 29;21(7):e1013336. doi: 10.1371/journal.pcbi.1013336 (PMC12316398; doi:10.1371/journal.pcbi.1013336)
Supplement: S4 File — (PDF) [file pcbi.1013336.s004.pdf]

Identifying the critical transition for bladder cancer

Critical states of BLCA

The graphical representation in **Figure S4A** indicates a sudden surge in the DNFE score at Stage II, implying a critical state or the potential onset of a critical transition. Following this transition, the distant metastasis of cancer initiates and there is a marked deterioration in the patients' tumor state. Figure S4B similarly discloses the critical state on the molecular plane, with the DNFE score demonstrating a significant rise at Stage II before indicating a progressive increase at Stage IV. Moreover, DNBs within the global or complete network display heightened sensitivity to the critical state prior to a deterioration in the disease. The prognostic analysis conducted is premised on the clinical data of BLCA samples and considers the survival times of samples both pre and post the crucial transition at Stage II. As manifested in Figure S4C, remarkable differences exist in the survival curves of BLCA before and after Stage II. Survival times for samples prior to reaching the critical state are noticeably longer compared to those post this stage. Figure S4D presents the DNB molecular network which is regulated by genes, revealing that the network structure in Stage II distinctly deviates from those in other stages. All these findings corroboratively establish Stage II as the critical state. Furthermore, Figures S4E and S4F illustrate that while utilizing the gene expressions of DNB genes alone cannot discriminate the critical state from other states, the application of DNFE scores of these DNB genes enables the detection of the critical state - a determination further supported by the information depicted in Figure S4A.
