# Supplementary figures and images for: DNFE: Directed network flow entropy for detecting tipping points during biological processes
Source: PLoS Comput Biol. 2025 Jul 29;21(7):e1013336. doi: 10.1371/journal.pcbi.1013336 (PMC12316398; doi:10.1371/journal.pcbi.1013336)

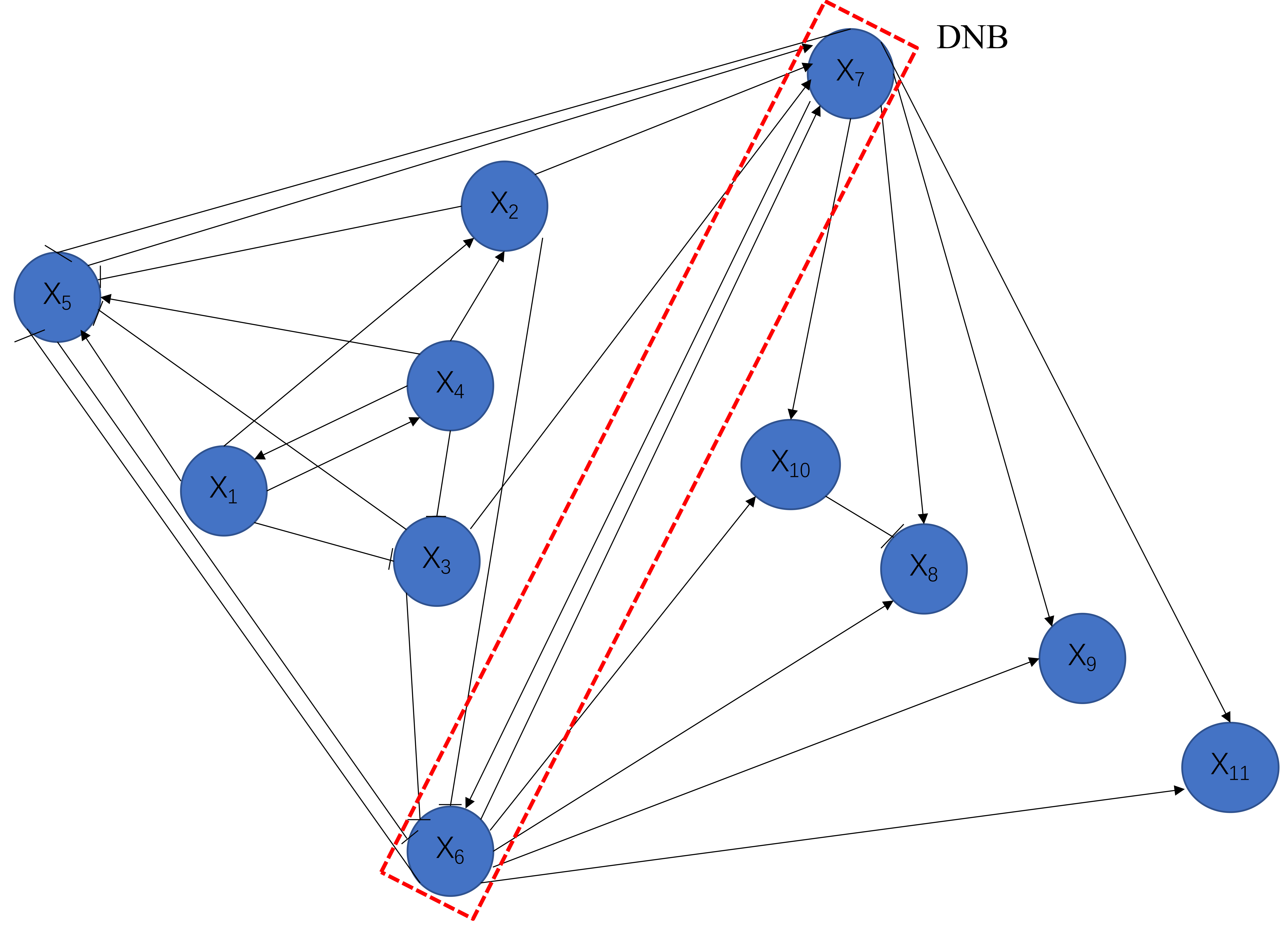

Supplement: S1 Fig — This illustration of a molecular network features eleven nodes, with their dynamic regulatory interactions defined based on the stochastic system in Eq. (S1) in S1 File. The positive or negative regulatory connections among the nodes are reflected by the edges. (JPG) [file pcbi.1013336.s005.jpg]

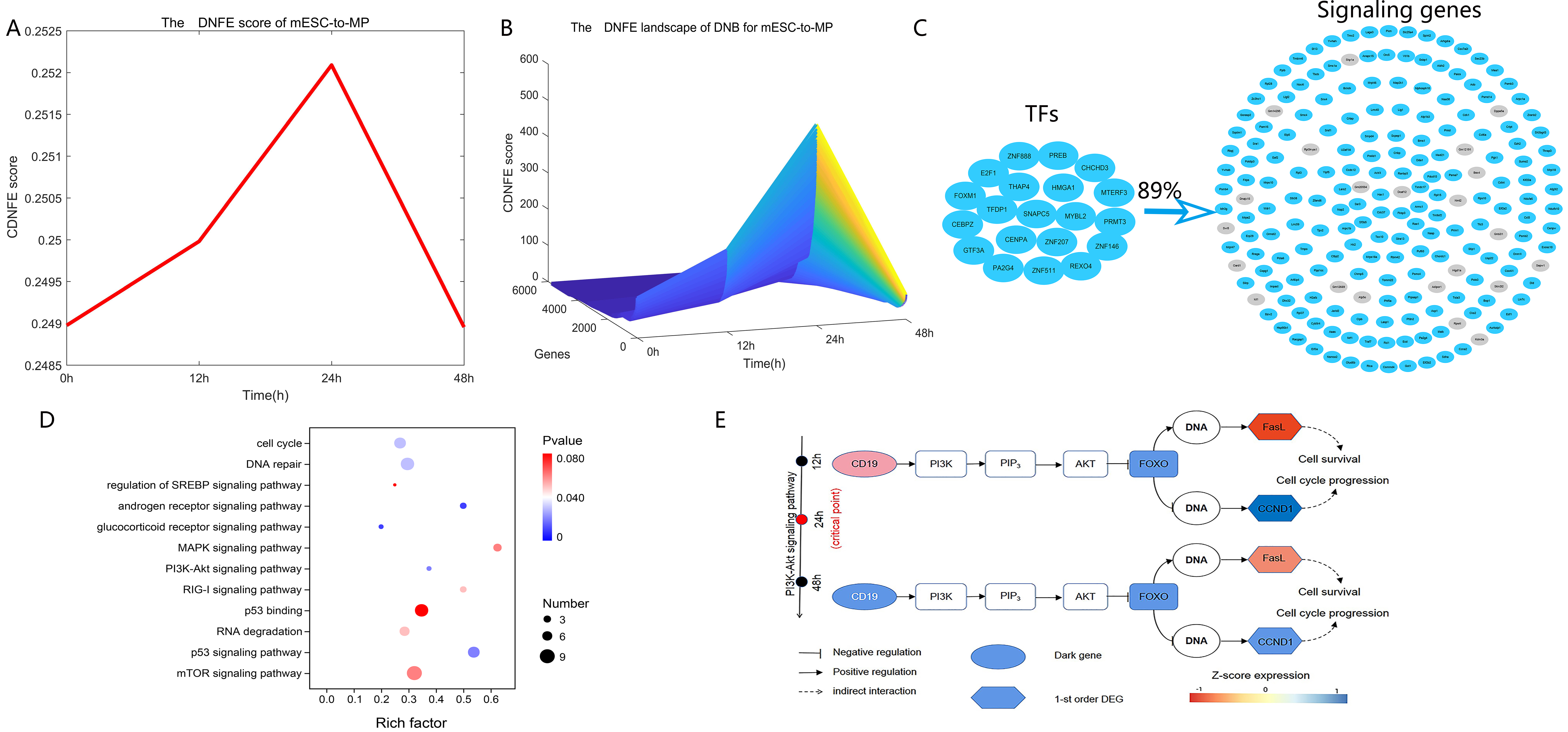

Supplement: S2 Fig — (A) The temporal fluctuations in the directed network flow entropy (DNFE) scores during the mESC-to-MP transition reveal the impending critical state at the 24 h mark. (B) The DNFE landscape for the mESC-to-MP transition demonstrates an apparent surge in the DNFE score at 24 h, underscoring its utility in pinpointing critical developmental stages. (C) The top 20 upstream hub transcription factors exert regulatory control over 89% of the dynamic network biomarker (DNB) genes detected at 24 h, highlighting the pivotal role of these factors in the gene regulatory network. (D) Analysis of Kyoto Encyclopedia of Genes and Genomes pathway enrichment for “dark genes” involved in the mESC-to-MP process. (E) The signaling genes within the PI3K/Akt pathway, which are enriched at the tipping point, exhibit distinct regulatory patterns before and after lymph node metastasis. These genes are instrumental in the progression of cancer. (JPG) [file pcbi.1013336.s006.jpg]

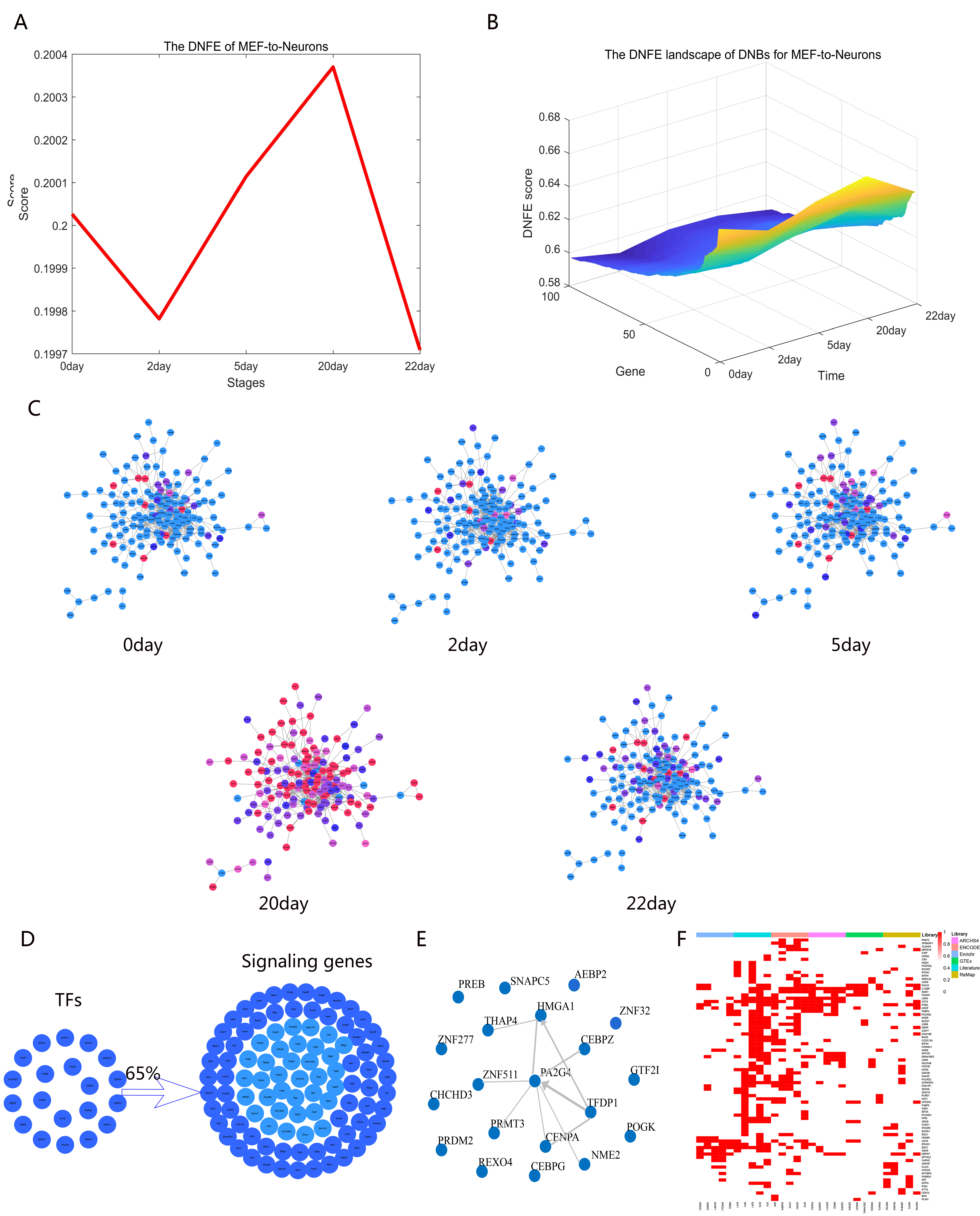

Supplement: S3 Fig — (A) The trajectory of the directed network flow entropy (DNFE) score throughout the MEF-to-neuron reprogramming timeline is marked by notable escalation at day 20, signifying a tipping point that heralds a critical phase in the cellular transition. (B) The DNFE landscape of dynamic network biomarkers (DNBs) during the MEF-to-neuron transition reveals a pronounced uptick in the DNFE score at day 20, which is indicative of a tipping point. (C) The temporal dynamics of DNBs throughout the MEF-to-neuron transition are characterized by a discernible pattern of change, with the critical state detectable at day 20. (D) Top 20 upstream hub transcription factors are able to regulate 89% of the identified DNB genes at day 20. (E) The results of the regulatory association between the top 20 transcription factors are shown as a network. (F) The top five transcription factors (TFs) identified using each library, with these TFs represented along the columns and the query genes along the rows. The entries within this matrix are populated based on whether a query gene is present or absent within the target gene set of a library TF at the second tipping point. (JPG) [file pcbi.1013336.s007.jpg]
