# Supplementary material for: DNFE: Directed network flow entropy for detecting tipping points during biological processes
Source: PLoS Comput Biol. 2025 Jul 29;21(7):e1013336. doi: 10.1371/journal.pcbi.1013336 (PMC12316398; doi:10.1371/journal.pcbi.1013336)
Supplement: S1 Table — (PDF) [file pcbi.1013336.s009.pdf]

Table 1 The comparison between the DNFE algorithm and other GRN methods.

**Methods**	**Dataset**	**Basis of Theory**	**Instability scores**	**Difference**
ANANSE	ChIP-seq/ATAC-seq /RNA-seq	A network-based method that uses properties of enhancers and their GRNs to predict key TFs in cell fate determination.	No	1.They constructed cell type-specific GRNs based on the inferred TF binding probability, the transcription factor activity, and the expression levels of the TF and predicted target genes.<br>2. For expression-based GRNs, they used only the mean of the scaled TPM of TFs and genes together as the interaction score of TFs and genes, which is stability score.<br>3. They calculated the **'influence score'**, a measure of importance of a TF in explaining transcriptional differences between two cell types.<br>4. Their method can be applied to ChIP-seq, ATAC-seq and RNA-seq.
Deepmaps	CITE-seq/ scRNA-seq/ scATAC-seq	A heterogeneous graph transformer framework for cell-type-specific biological network inference from scMulti-omics data.	No	1. They first constructed a matrix of cellular genes and then constructed a heterogeneous map.<br>2.**Attention scores** were generated to characterize the importance of cells to genes, which were stability score.<br>3. An HGT model is built to jointly learn the low-dimensional embedding for cells and genes and generate an attention score to indicate the importance of a gene to a cell.<br>4. Their method can also be applied to ChIP-seq, ATAC-seq and RNA-seq.
DNFE	Single-cell, bulk and body-fluid data	Directed network; Advanced topology structure of network; Network flow entropy; The critical slowing down theory; Bifurcation theory of dynamical systems; dynamic network<br>Biomarker theory	Yes	1. The DNFE method is proposed based on directed networks and calculates **DNFE scores** at different points in time or stages.<br>2.We designated 5% of the genes as DNB genes by their scores at different time points. The relationship between these genes and disease progression was found through enrichment analysis and more.<br>3. Our method can be applied to single-cell, bulk and body-fluid data.
