# Supplementary material for: DNFE: Directed network flow entropy for detecting tipping points during biological processes
Source: PLoS Comput Biol. 2025 Jul 29;21(7):e1013336. doi: 10.1371/journal.pcbi.1013336 (PMC12316398; doi:10.1371/journal.pcbi.1013336)
Supplement: S2 Table — (PDF) [file pcbi.1013336.s010.pdf]

Table 1 The detailed hallmark pathways enrichment analysis information

GeneSet	Description	pValue	FDR
hsa04024	cAMP signaling pathway	0.004728132	0.175100761
hsa03040	Spliceosome	0.022988506	0.219193163
hsa01212	Fatty acid metabolism	0.054669704	0.332226203
hsa05205	Proteoglycans in cancer	0.085910653	0.444525454
hsa05418	Fluid shear stress and atherosclerosis	0.052724077	0.465798608
hsa04145	Phagosome	0.057724957	0.50582733
hsa04510	Focal adhesion	0.075630252	0.50826972
hsa03010	Ribosome	0.094240838	0.513028169
hsa04217	Necroptosis	0.062913907	0.532693617
hsa02010	ABC transporters	0.115299335	0.544969398
hsa00071	Fatty acid degradation	0.134939759	0.547348485
hsa04921	Oxytocin signaling pathway	0.063356164	0.577330395
hsa04310	Wnt signaling pathway	0.304449648	0.590083926
hsa04066	HIF-1 signaling pathway	0.33568075	0.631250933
hsa05152	Tuberculosis	0.159132007	0.65554046
hsa04146	Peroxisome	0.372960372	0.656183759
hsa00010	Glycolysis / Gluconeogenesis	0.30024213	0.660615657
hsa05130	Pathogenic Escherichia coli infection	0.02915952	0.664498441
hsa04114	Oocyte meiosis	0.487931034	0.687237931
hsa00620	Pyruvate metabolism	0.244604316	0.71933
